# Identifying Novel DNA Adducts in Amphipods and Developing Sample Preparation for Adductomics Using Dispersive Solid-Phase Extraction

**DOI:** 10.1021/acs.est.5c02493

**Published:** 2025-10-30

**Authors:** Zareen Khan, Elena Gorokhova, Giulia Martella, Nisha H. Motwani, Natalia Tretyakova, Pedro F. M. Sousa, Hitesh V. Motwani

**Affiliations:** † Department of Environmental Science, 7675 Stockholm University, Stockholm SE-106 91, Sweden; ‡ School of Natural Sciences, Technology and Environmental Studies, Södertörn University, Huddinge SE-14189, Sweden; § Department of Materials and Environmental Chemistry, Stockholm University, Stockholm SE-106 91, Sweden; ∥ Department of Medicinal Chemistry and Masonic Cancer Center, 5635 University of Minnesota, Minneapolis, Minnesota 55455, United States

**Keywords:** DNA adducts, high-resolution mass spectrometry, nontarget analysis, dispersive solid-phase extraction, exposure assessment

## Abstract

The growing use of environmental DNA adductomics for exposure assessment emphasizes the need for improved methods that are adapted to chemically and biologically diverse samples. While liquid chromatography coupled to high-resolution mass spectrometry (LC–HRMS) has advanced DNA adductome analysis, sample cleanup methods remain challenging, especially in nonmammalian models. Here, we used amphipod *Monoporiea affinis*, a sentinel species in biological effect studies, as a surrogate crustacean species for the method development. It is particularly suitable because its chitinous tissues and high lipid content present a challenging matrix for DNA extraction. We addressed the following objectives: (i) to propose adduct structures by combining multiple tools for DNA adduct screening and (ii) to evaluate the applicability of dispersive solid-phase extraction (d-SPE) as a cleanup step for DNA adductome analysis. Toward the first objective, we integrated open-source software nLossFinder with a recently introduced DNA adductomics database (GitLab) to enhance the structural identification of unknown adducts. A combination of accurate mass data and MS2-fragmentation allowed differentiation of the complex mixture of nucleoside adducts, facilitating the structural identification of 16 DNA adducts, including 10 modifications on amphipod DNA reported for the first time. Toward the second objective, we introduced d-SPE as a novel cleanup approach for DNA adduct analysis in whole-body crustacean samples. Using Z-sep+ as the d-SPE sorbent, we demonstrated a major reduction of matrix interferences, including phospholipids, and enhanced sensitivity toward DNA adducts detection, leading to improved LC–HRMS signal response by up to 170%. d-SPE offers a simple alternative to conventional methods like standard SPE and liquid–liquid extraction, making it a valuable tool for DNA adductomics in environmental monitoring and aiding high-throughput capacity, especially while handling large numbers of samples. Further studies should include validation of this method for other species and DNA modifications. These advancements underscore the potential of the proposed data analysis workflow and d-SPE for improving mass spectrometry-based DNA adductomics in environmental monitoring, paving the way for more accurate and comprehensive exposure assessments across diverse species and environmental conditions.

## Graphical abstract

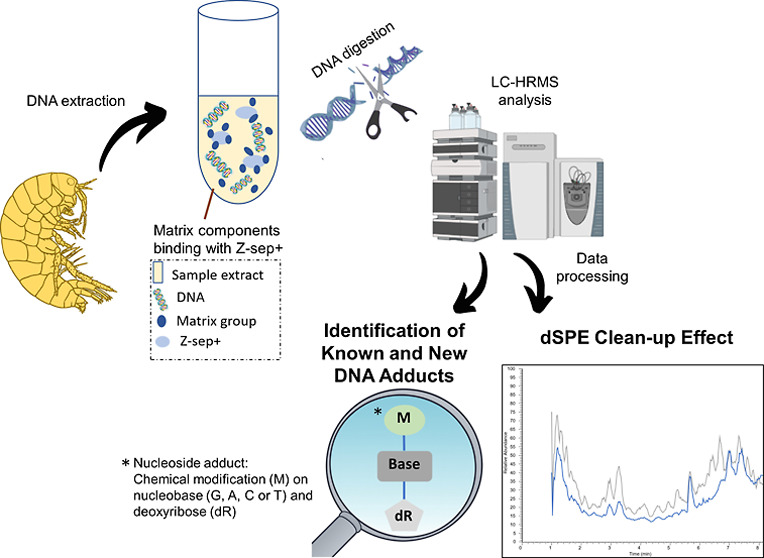

## 1 Introduction

DNA adductomics, an emerging field within the omics sciences, encompasses the comprehensive measurement of covalently bound chemical modifications on the nucleosides. The DNA modifications can be a result of environmental exposures to pollutants, as well as endogenous processes such as epigenetic regulation and the formation of electrophilic metabolites. The aim of DNA adductomics is to determine and characterize the totality of DNA adducts. Among common DNA modifications, epigenetic marks such as 5-methyl-2′-deoxycytidine (5-me-dC) and *N* ^6^-methyl-2′-deoxyadenosine (*N* ^6^-me-dA) are endogenously formed as a result of enzymatic activity of DNA methyltransferases and play a crucial role in regulating gene expression.^1,2^ Alternatively, adducts formed upon DNA oxidation by reactive oxygen species (ROS), e.g., 8-oxo-7,8-dihydro-2′-deoxyguanosine (8-oxo-dG), and adducts formed as a result of exposure to electrophilic agents [e.g., diol epoxide metabolites of benzo­[*a*]­pyrene, a polycyclic aromatic hydrocarbon] could be genotoxic.^3–6^ Complete characterization of the DNA adductome can contribute to identification of the sources of exposure and lead to an increased understanding of associated biological effects.^7^ Hence, the identification and accurate measurement of DNA adducts are crucial for various applications concerning exposure and effect assessment.

In environmental toxicology, DNA adducts can serve as biomarkers of exposure to electrophilic compounds/metabolites with prior research in fish and mussels^8,9^ focusing mainly on PAH-derived adducts. Unlike metabolomics, which assesses transient metabolic responses and intermediates of exposure (since metabolites often fluctuate rapidly and are cleared quickly), environmental DNA adductomics provides a more stable and integrative measure of exposure to potential genotoxicants.^10^ Covalent DNA modifications, if unrepaired, can persist over time, serving as a molecular archive of both acute and chronic exposures. Depending on the sampling approach, DNA adductomics can reflect systemic exposure, e.g., if whole organisms are analyzed or target dose of the DNA-modifying agent, e.g., if a specific tissue is dissected and analyzed.

Several analytical methods have been developed to measure DNA adducts including immunochemical, ^32^P-postlabeling, and mass spectrometry (MS) techniques.^11^ Recent developments in high-resolution mass spectrometry (HRMS) instrumentation have offered an approach that is effective for accurate and sensitive quantitation along with providing structural information for the modified nucleobases.^12^ HRMS has emerged to be the preferred analytical approach to overcome the major challenges of DNA adductome analysis, including broad range detection of DNA adducts from complex matrices^13,14^ and the detection and identification of unknowns in the absence of a relevant DNA adducts database.^15,16^

Structural identification of DNA adducts is a challenge for environmental DNA adductomics due to the complexity of the exposures and the paucity of databases of DNA modifications. For detecting unknown adducts in the post-MS data processing, our group has developed nLossFinder, a graphical user interface program available as an open-source.^15^ nLossFinder, based on the characteristic neutral loss of deoxyribose moiety (116.0474 Da), allows for the nontargeted detection of DNA adducts without any prior knowledge of their structures. In the present study, we explored the possibility of integrating the output from nLossFinder with a recently published DNA adducts database^17^ consisting of structural features and potential source of the adducts to aid in the identification and characterization of the detected adducts.

Another challenge in analyzing DNA modifications in environmental samples is ensuring that DNA is representative in the analyzed sample. Previous extraction methods for genomic studies based on chromatography favor high-quality DNA by removing fragmented molecules of DNA along with impurities such as lipids, proteins, and polysaccharides.^18,19^ However, mass spectrometry-based adductomics applications require isolation of high-quality DNA while avoiding the loss of fragmented DNA (as it might contain DNA adducts). In addition, when analyzing the DNA adductome, the quantity of biological material available for DNA extraction can be limited,^20^ such as from small invertebrates or specific tissues of larger animals. Moreover, samples prepared from whole-body homogenates, as often the case when analyzing small-bodied specimens, may contain high concentrations of cell components other than DNA, including hydrophilic endogenous metabolites, causing considerable matrix effects. In a typical LC–MS experimental workflow for DNA modifications analysis, DNA is digested to single nucleosides, followed by adduct enrichment. Common sample preparation methods for enrichment of DNA adducts from complex mixtures containing unmodified nucleosides include liquid–liquid extraction,^21,22^ ultrafiltration,^23^ off-line high-pressure liquid chromatography (HPLC),^24^ immunoaffinity chromatography,^25^ and solid-phase extraction (SPE).^26,27^ These techniques, however, have certain disadvantages;^20^ for instance, liquid–liquid extraction is limited to hydrophobic DNA adducts such as those from PAHs, not sufficient to recover low levels of DNA adducts, and requires a combination with other purification procedures. Ultrafiltration and off-line HPLC techniques suffer from relatively low throughput and limited specificity. Affinity chromatography is time-consuming, and there is limited availability and high cost of antibody-functionalized resins. While cartridge-based SPE methods can be useful for purification and enrichment of specific classes of DNA adducts due to their strong preconcentration capability, they are relatively labor-intensive and time-consuming compared to dispersive SPE (d-SPE). Moreover, in untargeted DNA adductomics, such selective enrichment carries the risk of losing modified nucleosides that are not retained, making d-SPE the more suitable approach.

d-SPE has been proposed as a superior sample cleanup approach compared to SPE due to its simplicity, eco-friendliness, and efficiency. It requires smaller amounts of sorbent and solvents, making it faster, more cost-effective, and reducing waste.^28,29^ All sorbent particles interact equally with the matrix, leading to a larger sorbent capacity per gram of sorbent. Different d-SPE cleanup strategies have been widely explored in, e.g., pesticide residue analysis.^30,31^ The crucial selection of an appropriate sorbent for d-SPE cleanup is pivotal to maximize matrix retention while minimizing analyte retention on the sorbent. Several previous studies have involved d-SPE cleanup using Z-sep during the analysis of a wide variety of compounds including pharmaceuticals,^32,33^ bisphenols,^34^ veterinary drugs,^35^ PAHs,^36^ and pesticide residues.^37^

We hypothesized that the addition of Z-sep+ as a d-SPE cleanup step would enhance the sensitivity of DNA adducts and decrease matrix effects, which would be particularly useful for complex environmental matrices, such as crustaceans that are notoriously difficult for efficient DNA extractions.^38^ The d-SPE technique was explored using the amphipod *Monoporiea affinis* as a model test organism. These amphipods are small sediment-living crustaceans used within the Swedish National Marine Monitoring Program (SNMMP) in the Baltic Sea to assess the biological effects of the environmental contaminants. Previous analyses of the DNA adductome in this species provided background knowledge of its composition.^10,39^ Moreover, this is the first study, to the best of our knowledge that has evaluated the use of d-SPE in the measurement of DNA adducts.

The aims of the present study were (i) to use the accurate mass data from LC–HRMS analysis for tentative structural identification of adducts in the amphipods, by combining nLossFinder for DNA adducts screening and an adductomics database and (ii) to evaluate the applicability of d-SPE as a cleanup step for DNA adductome analysis, in terms of the reduced matrix effect and enhanced LC–MS/MS analyte response. These developments are anticipated to have applications in tracing potential exposure sources for the identified adducts, improving sensitivity for adductomics analysis, and advancing the field of environmental adductomics.

## 2 Materials and Methods

### 2.1 Chemicals and Other Materials

Deoxyribonucleic acid from calf thymus (ctDNA) sodium salt, Z-sep+, 2′-deoxyguanosine (dG), 2′-deoxycytidine (dC), 2′-deoxyadenosine (dA), thymidine (T), 5-methyl-2′-deoxycytidine (5-me-dC), 8-oxo-7, 8-dihydro-2′-deoxyguanosine (8-oxod-G), *N* ^6^-methyl-2′-deoxyadenosine (*N* ^6^-me-dA), nuclease P1 (NP1) from *Penicillium citrinum*, phosphodiesterase I (SVPDE) from *Crotalus adamanteus* (snake) venom, alkaline phosphatase (AKP) from *Escherichia coli*, proteinase K, ammonium acetate, ammonium bicarbonate, Trizma hydrochloride solution (Tris-buffer, pH 7.4), zinc chloride, and formic acid were obtained from Sigma-Aldrich (St. Louis, MO). Chelex-100 resin was purchased from Bio-Rad (Solna, Sweden). All solvents used were of HPLC grade. DNA experiments were carried out in DNA LoBind tubes, 1.5 mL (Eppendorf).

### 2.2 Sample Collection and DNA Extraction

Amphipods (Crustacea, Amphipoda) used in this study do not come under any animal ethical regulations. The experiments were performed on *M. affinis* collected in the Northern Baltic Proper; the sampling details are described elsewhere.^39^ This amphipod is a small (6–8 mm), shrimp-like crustacean with a laterally compressed body, elongated antennae, and a curved tail. Only adult females were used here. The eggs, if present, were removed from the brood pouch using a microscope, and the females were immediately frozen at −80 °C until the DNA extraction.

The DNA extraction was carried out with Chelex-100, a chelating ion-exchange resin that binds to polar components of cells leading to cell lysis.^40^ The beads settle down along with the lysed cells, while the DNA remains in aqueous solution. Briefly, each amphipod was homogenized using a Kontec pestle and incubated with proteinase-K (600 mAU/mL, 15 μL) and Chelex solution (10% w/v, 450 μL) at 55 °C, 450 rpm for 8 h. After this, the mixture was centrifuged at 21,000 rcf for 10 min at room temperature. The supernatant was measured for DNA concentration (ng/μL) and purity ratio (*A*_260_/*A*_280_) using a Nanophotometer (Implen). An aliquot (200 μL) of the supernatant was transferred to another tube for d-SPE cleanup, and another aliquot (200 μL) of the same supernatant was used for DNA digestion without d-SPE cleanup.

### 2.3 Dispersive SPE for Sample Cleanup

Z-sep+, a silica functionalized with Zirconia and C18, was used as the d-SPE sorbent for sample cleanup. The adsorption of matrix interference on Z-sep+ involves the mechanism of Lewis acid–base interactions, where the Zirconia atom (on Z-sep+) acts as a Lewis acid (electron acceptor), while the phosphate or hydroxy functional group on lipids or fats, respectively, acts as a Lewis base (electron donor) (Figure S1). In addition, matrix components can also be removed by hydrophobic interactions with the C18 moiety of Z-sep+. In the cleanup step, fine powder of Z-sep+ (35 mg) was dispersed into the DNA extract obtained from each individual amphipod (200 μL, *n* = 9), followed by 2 min vortexing and 10 min centrifugation at 15,000 rcf at room temperature. The supernatant was collected, and DNA concentration (ng/μL) and purity ratio (*A*_260_/*A*_280_) were determined using a Nanophotometer (Implen).

### 2.4 Enzymatic DNA Digestion

The extracted DNA (15 μg/sample), from nine paired samples, each analyzed both before and after d-SPE cleanup, was digested as described earlier.^39^ Briefly, Tris-buffer (1 mM, pH 7.4) was added to arrive at a total volume of 300 μL. The digestion was initiated with the addition of ammonium acetate (0.1 M, 30 μL), zinc chloride (10 mM, 12 μL), and NP1 enzyme (0.1 U/μL, 12 μL). This mixture was incubated at 37 °C for an hour. The next step involved the addition of ammonium bicarbonate (1 M, 35 μL) and SVPDE I enzyme (0.000126 U/μL, 9.6 μL) and incubation at 37 °C for another hour. Subsequently, AKP enzyme (0.029 U/μL, 10.2 μL) was added and incubated at 37 °C for another hour. Finally, the mixture was centrifuged at 21,000 rcf (4 °C, 10 min). The supernatant containing the nucleosides was transferred to a septum-sealed vial for analysis by LC–MS. Potential interference from hydrolysis enzymes was negligible, given the centrifugation, chromatographic separation, and high-resolution accurate mass (HRAM) detection.

### 2.5 LC–HRMS/MS Method

A Dionex UltiMate 3000 LC device interfaced to an Orbitrap Q-Exactive HF mass spectrometer (Thermo Fisher Scientific, MA) with a heated electrospray ionization (HESI) source in positive ionization mode was used. An earlier developed LC gradient program^39^ was optimized for the ultraperformance liquid chromatography (UPLC) method using an Acquity UPLC high strength silica (HSS) T3 column (1.8 μm × 2.1 mm × 100 mm). The mobile phase consisted of a mixture of water–methanol: system A with 5% methanol, and system B with 95% methanol, each containing 0.1% formic acid. For the applied flow rate (200 μL/min), the LC gradient consisted of an initial equilibration at 5% B for 2 min, which was increased to 70% in 8 min and then to 100% in 2 min. After holding at this composition for 2 min, a ramping to the initial condition of 5% B was done in 1 min, and the system was re-equilibrated for 3 min before the next injection. The MS was run in full scan combined with data-independent acquisition mode, with the sheath gas, auxiliary gas, and sweep gas set at 30, 10, and 1 arbitrary units, respectively. The full MS scan was conducted at the resolution of 60,000, automatic gain control (AGC) target 3e^6^, maximum ion injection time (IT) 200 ms, and scan range from 200 to 350 *m*/*z*. The DIA sequential precursor windows method was set to a mass resolution of 30,000, AGC target 5e^5^, maximum ion IT 120 ms, loop count 16, and scan range from 195 to 355 *m*/*z*, which was divided into 16 discrete *m*/*z* ranges with an isolation window of 10 *m*/*z* (200 ± 5, 210 ± 5, and up to 350 ± 5 *m*/*z*). In DIA, the selected range of ions are sequentially isolated and fragmented, thus providing a more comprehensive data set of MS2-fragmentation compared to, e.g., data-dependent acquisition.^15^ See also Supporting Information, Note 1.

### 2.6 Identification of DNA Adducts

An in-house DNA adduct database (up to *m*/*z* 350) was established to reflect the predominance of low-molecular-weight adducts in *M. affinis*. The database was compiled from the data of cleaned samples combining literature reports,^10,39^ GitLab entries,^17^ and nontarget detection using nLossFinder,^15^ as described in Supporting Information (Note 2). Adduct candidates were first screened in TraceFinder, followed by manual evaluation in XCalibur to confirm MS1 and MS2 peaks at the same retention time with high mass accuracy (box 1, Note 2). Each entry included the compound name (or tentative assignment), chemical formula, accurate *m*/*z* of precursor and nucleobase fragment ions, and retention time under the applied LC conditions. The nLossFinder approach expanded the adductome by identifying putative adducts through the diagnostic neutral loss of deoxyribose (116.0474 Da), although redundant signals (isotopes and ESI adducts) were excluded after manual curation. This process yielded a curated database of 65 adducts (Table S1), which was imported into TraceFinder for sample screening. Adducts were considered detected when precursor and fragment ions were found within 5 ppm mass accuracy and at comparable retention times.

For structural identification, the analytes were evaluated for different levels of confidence based on the number of identification criteria that were met.^41^ First, the identities of 5-me-dC, *N* ^6^-me-dA, and 8-oxo-dG were confirmed using authentic reference standards by matching Rt and accurate mass of the molecular ion and corresponding nucleobase fragment ion as described previously.^39^ For the remaining 2′-deoxyribonucleoside adduct analytes, the following criteria of identification were met: (i) measured accurate *m*/*z* of the molecular ion within ±5 ppm mass accuracy of the corresponding calculated *m*/*z*; (ii) at least one HRMS/MS fragment ion (including from neutral loss) per compound with high mass accuracy (±5 ppm) at the same Rt as the molecular ion; (iii) Rt within ±0.2 min across all the test samples in the batch; (iv) an isotope pattern score of ≥80; and (v) presence of the compound in a minimum of 90% of the test samples across the batch.

### 2.7 Statistical Analysis

The statistical evaluation addressed whether there is a significant difference in DNA adducts between samples before and after d-SPE cleanup in *M. affinis* using a data set of DNA adduct profiles with 9 biological replicates, i.e., individual amphipods. Each sample had paired observations, i.e., with and without the d-SPE treatment. The data on the individual peak areas for the DNA adducts were log-transformed to stabilize the distribution and normalized by the corresponding dG peak area ([adduct peak area] × 100/[dG peak area]) to account for differences in DNA hydrolysis yield across samples. The pairwise comparisons for each adduct were conducted using the Nonparametric Wilcoxon Test based on the median rank scores. Then, a permutational multivariate analysis of variance (PERMANOVA) was used to evaluate the overall response of the adduct profile to the treatments followed by the Permutation test for homogeneity of multivariate dispersions (PERMDISP).^42^ In the multivariate testing, Bray–Curtis distances with 1000 Monte Carlo permutations were applied, as implemented in the PERMANOVA + add-on package for PRIMER+ v.7.^43^ The results of PERMANOVA were illustrated by a principal coordinate analysis (PCoA) plot.

## 3 Results

### 3.1 Identification of DNA Adducts in Amphipod Samples

Table 1 displays the putative DNA adducts identified in the amphipods with a high mass accuracy. The DNA adducts are arranged in the table as follows: A1 to A3: reference standards available; A4 to A6: structures elucidated from our previous publication;^39^ A7 to A16: structures elucidated from the GitLab database; A17 to A21: nonannotated *m*/*z* from our previous publication;^39^ and A22 to A32: nonannotated *m*/*z* from nLossFinder data processing.

**Table 1:** DNA Adducts Identified in Amphipods and Enhanced Analytical Response Following d-SPE Cleanup^a^

adduct number	chemical name	short name	Rt (min)	molecular ion *m*/*z* (MS1)	fragment ion *m*/*z* (MS2)	% difference before and after cleanup as median
A1	5-methyl-2′-deoxycytidine	5-me-dC	2.70	242.1135	126.0664	+67
A2	*N* ^6^-methyl-2′-deoxyadenosine	*N* ^6^-me-dA	4.50	266.1257	150.0777	+67
A3	8-oxo-7-8-dihydro-2′-deoxyguanosine	8-oxo-dG	5.47	284.0989	168.0518	+52
A4	5-hydroxy-2′-deoxycytidine	5-OH-dC	5.40	244.0932	128.0457	+123
A5	2′-deoxyinosine	dI	4.70	253.0931	137.0461	–43
A6	2′-deoxyuridine	dU	3.80	229.0818	113.0348	–27
A7	*N* ^4^-hydroxymethyl-5-methyl-2′-deoxycytidine	*N* ^4^-OHme-5-medC	1.80	272.1241	156.0768	+36
A8	methyl-glycol-2′-deoxycytidine	Me-glycol-dC	1.80	276.1190	160.0717	+56
A9	hydroxy-etheno-2′-deoxycytidine	OH-ε-dC	4.66	270.1090	154.0617	+44
A10	heptenal-2′-deoxycytidine	heptenal-dC	5.05	340.1872	224.1349; 298.1397^b^	+24
A11	dihydro-2′-deoxyadenosine	di-H-dA	3.87	254.1248	138.0755	+115
A12	*N* ^4^-(4-hydroxy-butyl)-2′-deoxycytidine	*N* ^4^-(4-OHbut)-dC	6.24	300.1559	184.1086	+29
A13	dihydro-2′-deoxyguanosine	di-H-dG	4.60	270.1202	154.0729	+109
A14	*N* ^2^-hydroxy-methyl-2′-deoxyguanosine	*N* ^2^-OHme-dG	3.79	298.1151	182.0678	+170
A15	5,6-dihydro-thymidine	5,6-di-H-dT	4.32	245.1137	129.0664	+30
A16	*N* ^6^-hydroxy-methyl-2′-deoxyadenosine	*N* ^6^-OHme-dA	5.40	282.1202	166.0729; 136.0617^b^	+61
A17	-	-	11.6	289.1758	173.1288	–6.3
A18	-	-	3.14	239.1139	123.0667	+38
A19	-	-	3.92	300.1299	184.0832	+151
A20	-	-	3.95	306.0603	190.0129	+57
A21	-	-	4.68	327.0504	211.0029	+30
A22	-	-	4.65	244.1005	128.0535	+159
A23	-	-	3.13	254.1151	138.0680	+71
A24	-	-	5.40	342.0140	225.9670	NF
A25	-	-	1.75	231.1339	115.0867	+47
A26	-	-	4.71	266.0822	150.0357	+160
A27	-	-	5.92	261.1302	145.0841	+42
A28	-	-	3.13	253.1117	137.0647	+40
A29	-	-	11.1	240.1593	124.1120	–2.9
A30	-	-	6.40	270.1632	154.1158	+29
A31	-	-	5.03	288.1916	172.1444	+43
A32	-	-	11.6	250.1187	134.0715; 119.0606^b^	+75
	2′-deoxyadenosine	dA	3.81	252.1094	136.0620	+51
	2′-deoxyguanosine	dG	4.64	268.1033	152.0568	+45
	2′-deoxycytidine	dC	2.11	228.0978	112.0507	+56
	thymidine	T	5.40	243.0971	127.0504	+96

a The adducts are given an arbitrary adduct number sequentially. This is followed by chemical and short name for the identified adducts. Adduct characterization by retention time (min), molecular ions detected (MS1, corresponding to the 2′-deoxyribonucleoside adduct), fragment ions detected after neutral loss (MS2, corresponding to the nucleobase adduct), and % difference in peak area before and after cleanup are included (+ or −, corresponding to % increase or decrease, respectively, after cleanup); all compounds were detected at high mass accuracy (within 5 ppm).

b Additional MS2 fragment ion detected. NF; not found in samples before cleanup. Empty space (-) under chemical and short name indicate adducts with no proposed chemical structure.

Figure 1 presents the proposed structures of all the identified adducts with the chemical formulas of the 2′-deoxyribonucleoside adducts and their calculated *m*/*z* for the MS1 molecular ion. Adducts A1 to A6 and A17 to A21 were previously reported.^10,39^ Among them, the identities of 5-me-dC (A1), *N* ^6^-me-dA (A2), and 8-oxo-dG (A3) were confirmed with the highest level of confidence (level 1) because of their match with corresponding reference standards in terms of Rt (min), MS1 (accurate mass of the nucleoside adduct molecular ion), and MS2 (nucleobase adduct fragment ion). A7 to A16 were identified for the first time in amphipods, to the best of our knowledge. These new adducts, for instance, *N* ^4^-OHme-5-medC (A7), Me-Glycol-dC (A8), OH-ε-dC (A9), and Heptenal-dC (A10), were identified based on their MS1, MS2, and isotopic pattern matched with their respective theoretical values. The structures proposed for these adducts are based on comparison of the accurate mass with the GitLab database.

**Figure 1:**
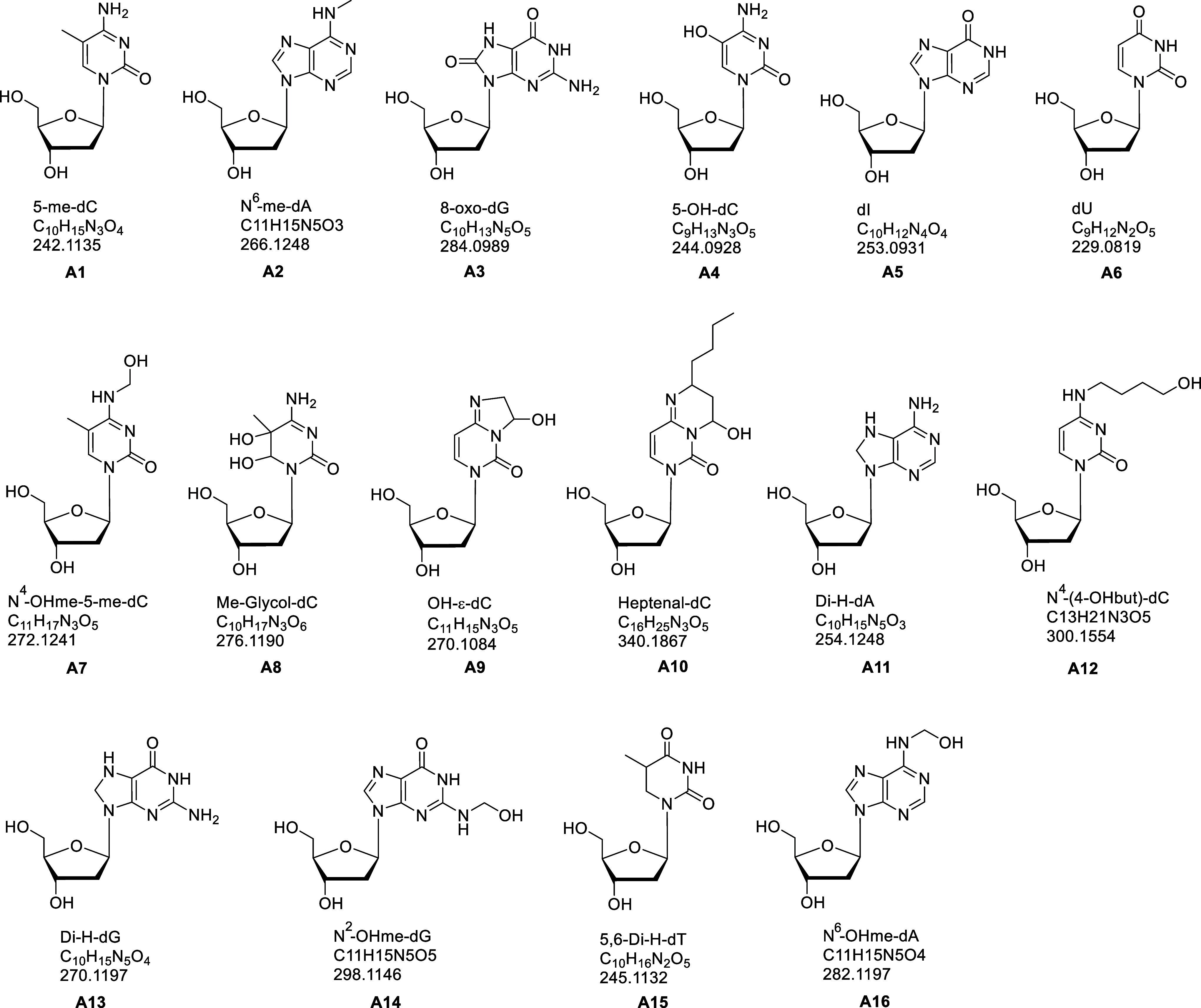
Structural information on DNA adducts identified in the amphipods. Proposed chemical structure, elemental composition, and calculated *m*/*z* [M + H]^+^ of the 2′-deoxyribonucleoside adducts (A1 to A16). The identification of 5-me-dC, *N* ^6^-me-dA, and 8-oxo-dG was confirmed by comparison with respective standards; the proposed structures of the others are based on the HRAM data and comparison with the GitLab database.

Even though the chemical composition determined has high confidence because of the use of HRAM data, since targeted MS2 was not performed, the position of the groups on the nucleobase should be considered as tentative. The nucleobase fragments (G, A, C, T as charged species from respective adduct analytes), resulting from the loss of both deoxyribose and the chemical modification, were not detected [except for *N* ^6^-OHme-dA (A16, A^+^ seen at *m*/*z* 136.0617)], probably because the collision energy was primarily optimized to give only the fragment resulting from the neutral loss of the deoxyribose. Additionally, putative adducts from nLossFinder, i.e., A17 to A32, were characterized based on their MS1 and MS2, present at the same Rt; however, no information about their annotation and chemical formula/structure was postulated.

### 3.2 Effect of d-SPE on Analyte Signals

#### 3.2.1 d-SPE Improved Analyte Signals

A substantial increase in LC–HRMS peak response (grand mean 60%) was observed, with 32 out of 36 adducts having significantly higher peak area values following the sample cleanup (*n* = 9 per group; Table 1). Adducts that showed maximum improvement in signal after d-SPE (% difference after cleanup in parentheses) include 5-OH-dC (+123), Di-H-dA (+115), Di-H-dG (+109), and *N* ^2^-OHme-dG (+170). On the other hand, a couple of them showed reduced peak areas after cleanup including dI (−43) and dU (−27).

d-SPE-based improvement is exemplified by the representative EIC of 5-me-dC (Rt 2.7 min) and *N* ^6^-me-dA (Rt 4.52 min), each exhibiting over a 50% increase in peak area and intensity (Figures S2A,B). Interestingly, the total number of adducts before and after d-SPE cleanup was the same, except one adduct (A24) not detected prior to d-SPE. Further, peak picking by TraceFinder was more accurate compared to that without d-SPE, which was attributed to the cleaner samples with reduced background signals. Therefore, our findings indicate that d-SPE cleanup enables a reliable automated data processing workflow, ensuring accurate results, particularly in peak integration. This can substantially save time that would otherwise be required for manual investigation and correction.

#### 3.2.2 Impact of d-SPE Cleanup Timing (before vs after Digestion)

To evaluate the application of d-SPE cleanup at the correct stage in the experimental workflow, we performed a test on the addition of Z-sep+ to the DNA extract before and after digestion. A reduction of peak response (50 to 78%) was observed for all the compounds in the case of the d-SPE applied after digestion for *M. affinis* compared to that applied before digestion. This is demonstrated by the representative EIC of 5-me-dC, *N* ^6^-me-dA, and *N* ^6^-OHme-dA, each exhibiting over a 70% decrease in peak area and intensity on application of d-SPE after digestion (Figure S3). Similar trend was observed for other adducts identified (Table S2). These results demonstrate the advantage of incorporating the d-SPE step immediately after DNA extraction compared to that after DNA digestion.

### 3.3 d-SPE Improved DNA Purity and Reduced LC–MS Matrix Peak Signals

Visually, DNA extracts appeared clear and nonturbid after d-SPE cleanup compared to untreated samples (Figure S4), likely due to the removal of phospholipids, fats, and pigments. Nanophotometer analysis confirmed significant differences in DNA purity and concentration following d-SPE cleanup (paired *t*-test, Figure S5). The *A*_260_/*A*_280_ ratio increased significantly (*p* = 0.024, t = 2.687, df = 8), indicating improved sample purity through removal of large-molecule contaminants. In contrast, DNA concentration decreased (*p* < 0.0001, t = 14.07, df = 8), which may reflect removal of fragmented DNA as well as matrix components absorbing at 260 nm. A pilot experiment with ctDNA supported this interpretation: DNA recovery after Z-Sep+ treatment exceeded 94%, with only a minor but significant (2–6%) reduction in two out of three replicates (Table S3), consistent with the selective removal of fragmented DNA and impurities while preserving intact DNA.

LC–HRMS analysis demonstrated substantial reduction of matrix interferences after d-SPE cleanup. Overlaid TICs showed a marked reduction of high-intensity background signals (Figure S6), particularly in the 2–7 min window where most adducts elute and are otherwise prone to suppression. In amphipod extracts (*n* = 9 per group), matrix signals were significantly reduced after cleanup. Evaluating the region of reduced matrix interferences (Figure S6), the signal of a dipeptide, Val–Leu “VL”, was almost eliminated (reduced by >98%). “VL” was identified at Rt 5.23 min with precursor mass (*m*/*z* 231.1703), matching elemental composition (C_11_H_23_O_3_N_2_), and the presence of multiple fragment ions, viz., *m*/*z* 72.0814, 86.0969, and 132.1019 (Figure S7). This identification agreed with that reported earlier.^44^ All the identified peptides including Leu–Leu “LL” (*m*/*z* 245.1859) and Ser–Leu “SL” (*m*/*z* 219.1340) were significantly reduced (up to 70%) after cleanup, as demonstrated by the paired *t*-test results (Figure S8). Thr–Leu “TL” (*m*/*z* 233.1492) was a dipeptide that did not show significant decrease post cleanup.

Beyond peptides, multiple phospholipids (e.g., *m*/*z* 553.2616, 490.2655, 479.7199, 480.2122) and fatty acyl/glycerolipids such as NAE 9:0 (*m*/*z* 202.1804), NATau 11:0 (*m*/*z* 294.1728), and MG 18:1 (*m*/*z* 374.3263) were significantly reduced (Figure S9). Conversely, one glycerophospholipid (*m*/*z* 532.2801) increased in abundance, likely due to reduced ion suppression or altered ionization efficiency after removal of competing matrix components. Overall, the depletion of peptides and phospholipids—major interferents in LC–MS^45^ and abundant in marine animals (20–70%) such as *M. affinis* ^46^—demonstrates the effectiveness of d-SPE cleanup in reducing matrix interferences.

### 3.4 d-SPE Effects on Relative Abundances of Specific DNA Adducts

The consistent increase in LC–MS signals for most DNA adducts following d-SPE cleanup led to improved accuracy and sensitivity of their measurement. The Wilcoxon-signed rank test was applied to assess the fold change in DNA adduct levels following cleanup treatment, and the results are summarized in Figure 2 and Table S4. Most DNA adducts displayed significant positive changes (*p* < 0.01) in their median fold levels, as indicated by consistently positive signed ranks and discrepancies. Conversely, two adducts exhibited significant decrease, namely, dI (median = 0.566, *p* = 0.004) and dU (median = 0.737, *p* = 0.04). Two adducts, A17 (*m*/*z* 289.1758) and A29 (*m*/*z* 240.1593), showed no significant changes, with discrepancies close to zero and overlapping confidence intervals.

**Figure 2:**
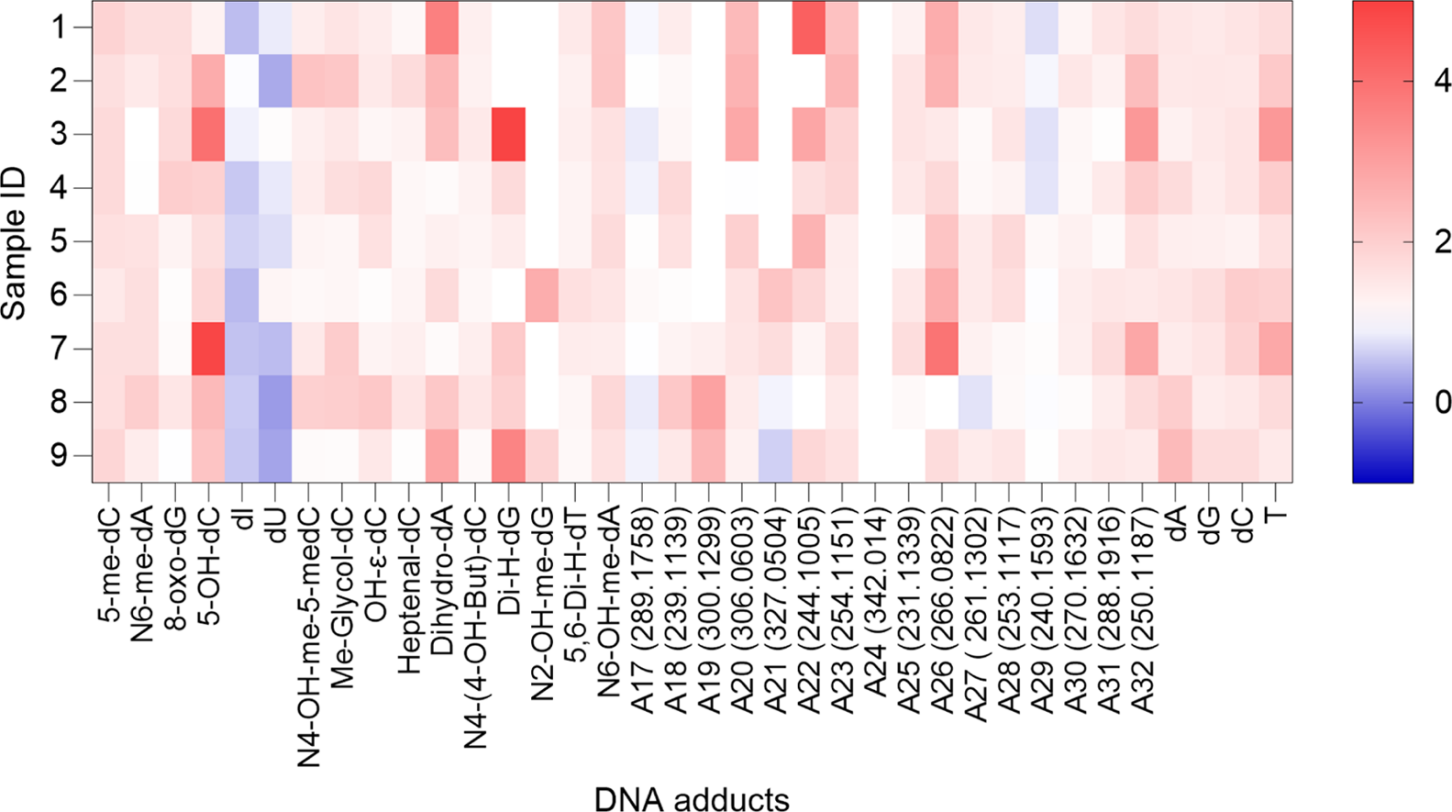
Fold change in DNA adduct peaks after d-SPE cleanup treatment. The heatmap displays the relative changes in adduct intensity following d-SPE treatment. Color intensity reflects the magnitude of fold change, with darker shades of red indicating higher values. Rows represent individual samples, and DNA adducts are shown on the horizontal axis. The significance of the change was analyzed by the Wilcoxon-Signed Rank Test, with 1 (white) denoting no change. In all cases, the increase in DNA adduct response post cleanup was significant, except for dI, dU, A17 (*m*/*z* 289.1758), and A29 (*m*/*z* 240.1593).

The cleanup treatment significantly increased nucleoside adduct response in both absolute and normalized data sets, as reflected by the reduced dispersion and clear group separation observed by the multivariate analysis. The effect of treatment and normalization on DNA adduct levels was evaluated using PERMANOVA, PERMDISP, and Principal Coordinate Analysis (PCoA) for both absolute peak area values and values normalized to dG. For the absolute peak area values, the treatment was highly significant (PERMANOVA; pseudo-*F* = 30.019, p = 0.001), with a component of variation of 4.6922 (square root = 2.1662) and a clear separation between the treatment groups along PCO1, which explained 77.3% of the total variation (Figure 3A). Moreover, we found significant differences in dispersion between groups (PERMDISP; *F* = 40.251, p = 0.001) with the no-cleanup group showing greater variability (mean distance to centroid = 1.4327 ± 0.1212) compared to the Z-sep+ group, which was more tightly clustered (0.6398 ± 0.0304).

**Figure 3:**
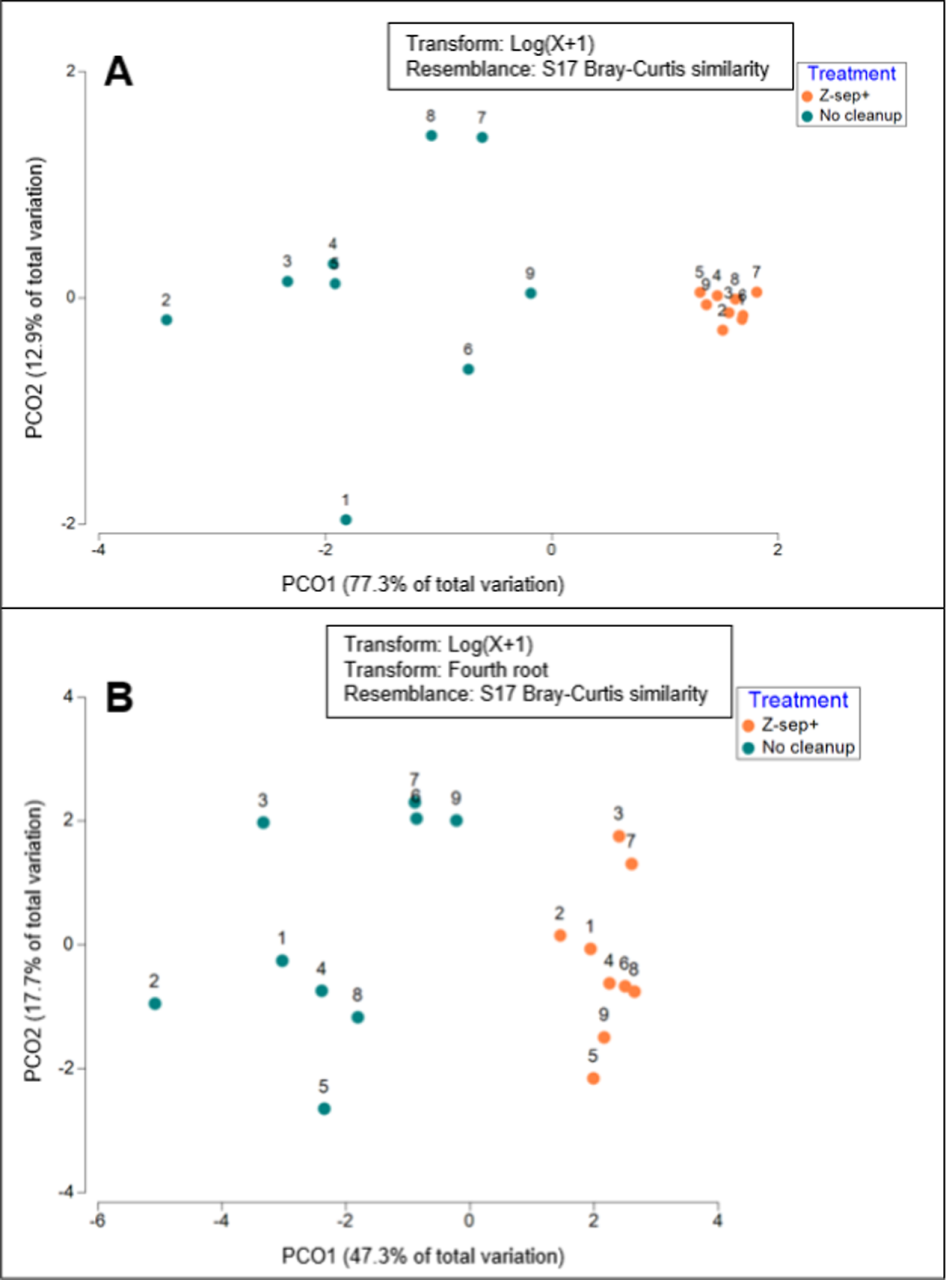
Principal Coordinate Analysis (PCoA) of DNA adduct levels (A, absolute peak area values; and B, normalized to dG values). PCoA plot showing the separation between untreated samples (no cleanup, teal) and treated samples (Z-sep+, orange) based on DNA adducts presented in Table 1. The first principal coordinate (PCO1) explains 77.3% of the total variation, while PCO2 accounts for 12.9%. Untreated samples exhibit greater dispersion compared to the treated group, which is tightly clustered, reflecting reduced variability following cleanup treatment.

For the normalized values, treatment also had a significant effect, but the pseudo-*F* value decreased to 10.707 (*p* = 0.001) compared to the absolute peak values. The component of variation increased to 9.1935 (square root = 3.0321), indicating an amplified absolute difference between the groups after normalization. PERMDISP again showed significant differences in dispersion (*F* = 39.025, *p* = 0.001), with greater overall variability in the normalized data set. The mean distance to the centroid increased to 3.25 ± 0.16 for the untreated group, while the cleaned-up group retained lower variability (2.06 ± 0.10). The PCoA plot for normalized values (Figure 3B) showed group separation primarily along PCO1 (47.3% of the total variation) and PCO2 (17.7%). Thus, the treatment effect remained robust in the normalized data set, with a smaller relative treatment effect but an increased magnitude of contribution for key adducts, and consistently reduced within-group dispersion, as reflected by the clustering observed in both data sets.

Additionally, SIMPER analysis was performed to identify individual adducts’ contribution to the treatment effect in the normalized data set. The adducts contributing most prominently to the 70% differences between the untreated and treated groups are shown in Figure S10. Adducts in the gray-shaded area made the largest contributions, with their normalized levels usually lower in the untreated group compared to the treated group.

## 4 Discussion

In this study, we demonstrated that combining d-SPE cleanup with LC–HRMS analysis and a curated in-house adductomics database enables improved identification and profiling of DNA adducts in amphipods. The workflow allowed identification of 32 DNA adducts, including several not previously reported in this species (e.g., heptenal-dC and dihydro-dA; Table 1), while effectively reducing matrix interferences, such as peptides and phospholipids. This enhanced signal quality (24–170%) and detection for most adducts, thereby facilitating automated data processing and improving comparability across samples. These findings support the use of d-SPE as a simple and broadly applicable pretreatment that increases analytical sensitivity and consistency in environmental DNA adductomics.

### 4.1 Interpreting Sources of the Identified DNA Adducts

Based on their structure (Figure 1), the adducts can be broadly attributed to three categories of processes: (i) endogenous formation of ROS, lipid peroxidation (LPO), and reactive nitrogen species (RNS), (ii) alkylation by electrophilic agents, either directly or via metabolic activation followed by binding with DNA, and (iii) endogenous DNA methylation due to epigenetic changes. Most of the detected modifications appear to arise from oxidative DNA damage, such as 8-oxo-dG (A3), 5-OH-dC (A4), Di-H-dA (A11), Di-H-dG (A13), and 5,6-Di-H-dT (A15). ROS, a byproduct of cellular metabolism, interact with cellular biomolecules, including DNA,^47–49^ generating these modifications. ROS also promote lipid peroxidation, and the resulting LPO end products^50–52^ may covalently bind with DNA, explaining the presence of LPO-derived adducts, such as Me-glycol-dC (A8), OH-ε-dC (A9), and heptenal-dC (A10). In addition, dI (A5) and dU (A6) are likely products of RNS interaction with dA and dC, respectively.^53^

We also identified adducts suggestive of alkylation, including *N* ^4^-OHme-5-medC (A7), *N* ^4^-(4-OHbut)-dC (A12), and *N* ^2^-OHme-dG (A14). Their possible precursors, such as formaldehyde to *N* ^4^-OHme-5-medC and *N* ^2^-OHme-dG^54,55^ and *N*-nitrosopyrrolidine to *N* ^4^-(4-OHbut)-dC (via metabolic activation),^56^ are known sediment contaminants,^57,58^ suggesting that exposure followed by biotransformation may explain their occurrence. However, to establish such an exposure–adduct relationship in the amphipods, controlled experimental studies are needed. Further, although the elemental composition of these adducts was determined from HRAM MS1 data and a corresponding nucleobase adduct fragment ion was detected in MS2, the exact positions of the chemical modifications cannot be confirmed without a comparison to reference standards. These findings therefore highlight the need for further structural confirmation.

Finally, the epigenetic marks, including 5-me-dC (A1) and *N* ^6^-me-dA (A2), are formed by DNA methylation on 5 C of dC and *N* ^6^ of dA, respectively, catalyzed by DNA methyltransferases and using *S*-adenosylmethionine as the methyl donor.^2^ In addition, we observed *N* ^6^-OHme-dA (A16), which may arise either as an oxidative transformation product of *N* ^6^-me-dA^59^ or via alkylation by formaldehyde.^54,55^

### 4.2 Improved Sample Preparation Workflow

We propose a workflow that integrates d-SPE as a purification step following Chelex-based extraction in DNA adductomics analysis (Figure 4). Chelex (an ion-exchange resin) was chosen over phenol–chloroform or kit-based procedures. Phenol–chloroform extraction, although historically common, is increasingly avoided due to concerns over toxicity and handling, while kit-based procedures yield high-quality DNA but are relatively complex and costly. Chelex, by contrast, provides higher yields that contain a mixture of unfragmented and fragmented DNA, which is advantageous for adductomics, since adducts may be present on fragmented molecules. In addition, Chelex extracts contain both DNA and RNA, making them useful for potential downstream multiadductomics analysis.^16^ The efficacy of Chelex in genomic applications has also been demonstrated.^60,61^

**Figure 4:**
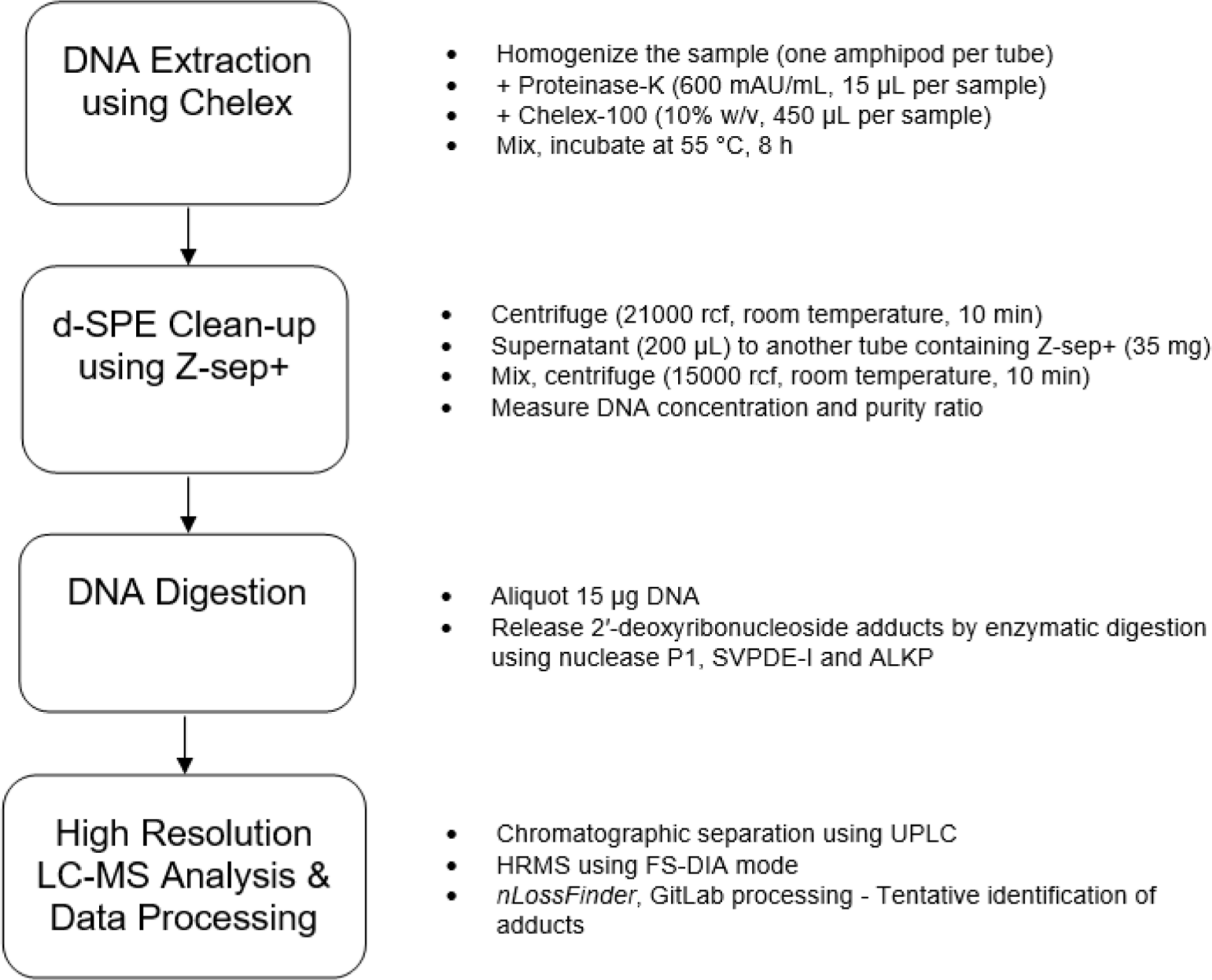
Workflow for DNA adducts analysis showing sample preparation steps: Chelex-based extraction, d-SPE cleanup using Z-sep+, and DNA digestion, followed by LC–HRMS analysis. The integration of d-SPE cleanup step after DNA extraction and prior to DNA digestion was favorable for enhanced DNA adducts sensitivity. Concentrations and volumes are given per sample. The data processing strategy was improved using nLossFinder and GitLab DNA adducts database.

A drawback of the Chelex method is that extracts also contain a multitude of matrix coextractives, such as phospholipids and fats, which can interfere with LC–MS analysis, motivating a follow-up purification step. By applying d-SPE after Chelex extraction, we efficiently removed these coextractives while retaining DNA integrity, thereby improving the quality of the material for downstream adductomic profiling.

### 4.3 Advantages of d-SPE Compared to Conventional SPE

Compared with conventional SPE, d-SPE offers important advantages for DNA adductomics. Conventional SPE is effective for selective enrichment of known adduct classes; for example, octadecylsilyl (C18) and amino (NH_2_) columns have been used to enrich hydrophobic and hydrophilic adducts, respectively.^26,27^ However, this targeted nature limits SPE in untargeted applications, where prior knowledge of adduct structures is lacking. In contrast, d-SPE removes interfering matrix components without bias toward specific adduct classes, thereby enhancing the detection and sensitivity of a broad spectrum of modifications. In this study, although we focused on low-molecular-weight DNA adducts (<350 *m*/*z*), the approach encompassed ∼270 adducts from diverse sources such as LPO, ROS, RNS, alkylation, etc.^17^

Interestingly, only two nucleosides, dI and dU, showed reduced peak responses after d-SPE cleanup, whereas all other nucleosides were either unaffected or enhanced. This selective reduction can be rationalized by the possible presence of free dI and dU in the tissue extract and their interaction with the Z-Sep+ sorbent upon d-SPE cleanup (Figure S1). Both dI and dU contain oxygenated functional groups (hydroxyl and carbonyl moieties) that may interact with zirconia sites similarly to phosphate or hydroxyl groups in matrix constituents, leading to partial retention on the sorbent and, thus, the observed decrease in dI and dU signals. Given that d-SPE consistently improved cleanup across nearly all detected adducts (Section 3.2) and is applied as an upstream procedure, similar benefits are expected for bulky DNA adducts (*m*/*z* > 350), supporting the broad applicability of d-SPE.

The timing of the d-SPE application was also critical. In earlier studies, SPE has been employed for purification of 2′-deoxyribonucleoside adducts after DNA digestion and prior to LC–MS analysis;^26,27^ however, when applied postdigestion in our study, it resulted in a major reduction of adduct signal intensity. This likely reflects direct coordination of exposed hydroxyl groups of 2′-deoxyribonucleoside adducts with the zirconium ions on the Z-sep+ sorbent, causing analyte loss. Conversely, when d-SPE was applied predigestion, matrix components such as phospholipids, proteins, and fats preferentially bound to zirconium (via the hydroxyl or other polar functional groups) (Figure S1), while the intact DNA polymer interacted only minimally, preserving adduct integrity.

Thus, while SPE remains useful for targeted enrichment, d-SPE offers a simple, efficient, and broadly applicable cleanup strategy ideally suited for environmental adductomics, especially in nonmodel species where the diversity of adducts cannot be predicted in advance. Moreover, the high biochemical diversity of nonmodel organisms, particularly invertebrates with complex matrices rich in chitin, fats, and waxes, appears to be handled effectively by the d-SPE approach.

### 4.4 d-SPE Cleanup for Adductome Profile Consistency in a Population and Analytical Validity

Multivariate analysis of the DNA adductome profiles showed that the d-SPE reduced between-individual variability, producing more consistent adduct profiles among amphipods collected at the same site and thus exposed to similar environmental conditions and exposure levels. This likely reflects the removal of interfering biochemicals, such as phospholipids and peptides, which otherwise enhance matrix effects and variable ionization.

Such consistency is important for analytical validity and the application of biological effect markers in environmental assessment. Uniform measurements more accurately represent the exposure conditions being studied, facilitate comparisons across sites with different pollution loads, and identify trends. Low intrapopulation variability also reduces uncertainty and allows the establishment of background levels of adduct variability within a population. Moreover, when measurements are uniform, it is easier for other researchers to replicate the analysis and confirm the findings independently using the appropriate quality control statistics. These improvements allow impacts of environmental factors driving the adductome changes to be assessed directly, rather than obscured by measurement artifacts or requiring additional replicates to correct for intrapopulation variability.^62,63^

Normalization strategies further contribute to consistency by accounting for sample-specific variation in DNA digestion or ionization efficiency. We applied normalization using the relative peak area of dG from the same LC–HRMS run. This adjustment not only accounted for differences in DNA hydrolysis yield but also introduced complexity because the dG signal itself is affected by matrix-dependent ion suppression. Accordingly, in the PCoA plot (Figure 3), a greater spread between samples was observed after normalization compared to the non-normalized data, reflecting differences in matrix effects between cleaned and noncleaned extracts. LC–UV quantification of dG has been suggested as a more robust alternative because it is less susceptible to matrix effects.^64,65^ Thus, the normalization and cleanup are interconnected: by reducing matrix interferences, d-SPE not only improves raw adduct signal uniformity but also enhances the reliability of normalization. Future studies should therefore combine LC–UV-based dG quantification with LC–HRMS adductomics to provide a stronger normalization framework and improve comparability across samples and studies.

### 4.5 Implications for Method Development and Environmental Monitoring

Most advances in DNA adductomics have centered on MS-acquisition and data processing, while sample pretreatment has received relatively little attention. Yet, effective cleanup is critical for recovering trace analytes and minimizing matrix interferences. Here, we demonstrate that d-SPE provides a straightforward, cost-effective, and time-efficient method for removing interfering matrix components, such as phospholipids, while preserving a broad range of DNA adducts. The method integrates seamlessly into existing workflows and is particularly suited for high-throughput applications such as in environmental screening and monitoring surveys.

Applied to the amphipod *M. affinis*, d-SPE enhanced recovery across nearly all detected adducts, improved analytical sensitivity, and enabled a more comprehensive characterization of the adductome without alteration of relative patterns. Several novel adducts were tentatively identified based on accurate mass and MS fragmentation, although confirmation with reference standards is still required. Beyond the well-known epigenetic marks, the detected adducts likely arising from ROS, LPO, RNS, and alkylation highlight the potential to serve as biomarkers. Establishing causal links between contaminant exposure and specific adducts will, however, require laboratory-based exposure experiments, which can validate these structural assignments and clarify whether the detected modifications serve as reliable biomarkers of exposure.

Overall, the improved cleanup procedure contributes to an effective and generalizable workflow that strengthens DNA adductomics as a tool for assessing the exposome and genome modifications that are not captured by other omics platforms. By reducing matrix effects and improving consistency, d-SPE significantly decreases intrapopulation variability in adduct profiles, enabling the establishment of background levels of adduct variability within populations for defining thresholds in impact assessment. At the same time, the approach has a potential to manage the substantial biochemical diversity found across and within nonmodel organisms while preserving a broad spectrum of adducts. These advantages highlight the potential of d-SPE to support further standardization of DNA adductomics workflows across species. Such standardization would not only facilitate cross-taxa comparisons but also move the field one step closer to a One Health framework since DNA adducts are a common feature across all living organisms.

## Supplementary Material



## Notes

LC–HRMS approach, adductomics database, and figures on the d-SPE mechanism and effects of d-SPE cleanup (PDF)
