# Supplementary material for: Identifying Novel DNA Adducts in Amphipods and Developing Sample Preparation for Adductomics Using Dispersive Solid-Phase Extraction
Source: Environ Sci Technol. 2025 Oct 30;59(45):24233–45. doi: 10.1021/acs.est.5c02493 (PMC12631988; doi:10.1021/acs.est.5c02493)
Supplement: Supplementary file 1 [file es5c02493_si_001.pdf]

Supplementary Material

Identifying novel DNA adducts in amphipods and developing sample preparation for adductomics using dispersive solid phase extraction

Zareen Khan[1], Elena Gorokhova[1], Giulia Martella[1], Nisha H. Motwani[2], Natalia Tretyakova[4], Pedro F.M. Sousa[3], Hitesh V. Motwani[1,*]

[1]Department of Environmental Science, Stockholm University, SE-106 91 Stockholm, Sweden

[2]School of Natural Sciences, Technology and Environmental Studies, Södertörn University, SE-14189 Huddinge, Sweden

[3]Department of Materials and Environmental Chemistry, Stockholm University, SE-106 91 Stockholm, Sweden

[4]Department of Medicinal Chemistry and Masonic Cancer Center, University of Minnesota, Minneapolis, Minnesota 55455, United States

[*]Corresponding author: hitesh.motwani@aces.su.se

List of Contents	Page
**NOTES**	
**Note 1. LC-HRMS approach**	S4
**Note 2. DNA adductomics database**	S4
**FIGURES**	
**Fig. S1. Illustration of retention mechanism on Z-sep+ sorbent for matrix components exemplified with a phospholipid.** Zirconia (Zr) coated on silica acts as a Lewis acid, while the phosphate group of phospholipids (or hydroxy moiety of fats, such as monoacylglycerol) act as a Lewis base during the interaction.	S6
**Fig. S2. Improved signal of 5-me-dC (A) and N^6-me-dA (B) employing d-SPE clean-up in amphipod DNA followed by their spectral identification with parent ion (MS1) and fragment ion (MS2), both within 5 ppm mass accuracy**. In case of 5-me-dC, the EIC at observed *m/z* 242.1135 (± 5ppm) showing the signal in DNA extract processed without d-SPE clean-up (solid line) and the correspondingly enhanced signal intensity, approx. by 50% (dotted line) after use of d-SPE clean-up (dotted line) under similar processing conditions. Similarly, for N^6-me-dA the EIC at observed *m/z* 266.1250 (± 5ppm) was correspondingly enhanced by 70 % after d-SPE clean-up.	S7
**Fig. S3. Comparison of peak responses of 5-me-dC, N^6-me-dA** and **N^6-OHme-dA in extracts with no clean-up (A); clean-up before digestion (B) and clean-up after digestion (C).** Retention time (Rt, min) and peak area (AA) are indicated on each chromatogram. Clean-up performed prior to digestion markedly enhanced peak intensity, consistent with removal of interfering matrix components before enzymatic hydrolysis. In contrast, clean-up after digestion resulted in notable signal loss for all analytes.	S8
**Fig. S4. Visual comparison of the amphipod DNA extract before (left) and after (right) d-SPE clean-up**. The extract before clean-up, using 35 mg Z-sep+, was visibly more turbid compared to that after clean-up because of the potential removal of phospholipids, fats and pigment moieties from the extract.	S9
**Fig. S5. Paired t-test results showing changes in (A) A260/280 purity ratio and (B) DNA concentration (ng/µL) before and after d-SPE clean-up.** Significant increase in A260/280 ratio ($p < 0.024$, $t = 2.687$, $df = 8$) indicates improved sample purity, while significant decrease in DNA concentration ($p < 0.0001$, $t = 14.07$, $df = 8$) reflects removal of contaminants contributing to initial overestimation of DNA content.	S9
**Fig. S6. A section of overlaid chromatograms of the amphipod DNA digest following d-SPE extraction with Z-sep+ (continuous line) and without d-SPE (dotted line) demonstrating the effect of the clean-up on the removal of matrix co-extractives.** The relatively high intensity of numerous matrix background signals in the extract without clean-up could be effectively reduced after clean-up, especially at the retention time where most of the 2'-deoxyribonucleoside adducts elute (within the range of 2 to 7 min). Peaks representing peptides and a phospholipid are marked as * and ¤, respectively.	S10
**Fig. S7. d-SPE clean-up effect: Reduction of dipeptide signal from the matrix after optimized d-SPE clean-up.** Extracted ion chromatogram for *m/z* 231.1703 (± 5 ppm) showing the signal intensity of dipeptide 'VL' at Rt 5.23 min without d-SPE clean-up (**A**) and the correspondingly reduced signal intensity of 'VL' after d-SPE clean-up (**B**); **C**: Identification of 'VL' with respect to parent ion (MS1, and chemical formula) and fragment ions (MS2).	S11
**Fig. S8. Paired t-test results showing changes in peak areas for the dipeptides 'VL' 'LL' and 'TL' (*m/z* in parenthesis) before and after d-SPE clean-up as measured by LC-MS analysis.** 'VL' and 'LL' exist as isomers at different Rt. Significant decrease in peak areas of these matrix compounds (p value summary; * =level of significance), except for TL, indicates sample purification as a result of d-SPE clean-up effect.	S12

List of Contents	Page
**Fig. S9. Paired t-test results showing changes in peak areas for the phospholipid 1, phospholipid 2, phospholipid 3, glycerophospholipid, MG, NAE and NATau (*m/z* in parenthesis) before and after d-SPE clean-up as measured by LC-MS analysis.** The lipids were identified based on LIPID MAPS and MS-DIAL data processing. Significant decrease in peak areas of these matrix compounds (p value summary; * =level of significance), except glycerophospholipid, indicates sample purification as a result of d-SPE clean-up effect.	S13
**Fig. S10. Contributions of individual DNA adducts to the dissimilarity due to the treatment effect based on SIMPER analysis in the normalized dataset.** Bar plot showing the average values of the most influential DNA adducts and their contribution (%) to the dissimilarity between the untreated samples (No clean-up, teal) and the same samples after the treatment (Z-sep+, orange); the adducts shown on this plot contributed 70% of the dissimilarity between the groups. Adducts in the grey-shaded area represent the most influential contributors to the treatment effect, with reduced levels (except dI) in the untreated group compared to the Z-sep+ group.	S14
**TABLES**	
**Table S1. In-house DNA adduct database for screening of 2′-deoxyribonucleoside adducts in amphipods.** Compound name for identified DNA adducts given as their standard abbreviation and for unidentified adducts as *m/z* of the parent ion. For each compound, the target peak (ms1 $[M + H]^{+}$), fragment peaks (ms2 $[M+H -dR]^{+}$ and $[dR]^{+}$) as well as retention time (min) are provided. A1-A19 (in the database table S1) are referring to adduct masses with the same A# as reported earlier in amphipods (Gorokhova et al, 2020).39 They may not necessarily be the same number as that in Table 1.	S15
**Table S2. Comparison of peak responses (absolute signal intensities) of representative nucleosides and DNA adducts in samples with no clean-up, d-SPE clean-up before digestion, and d-SPE clean-up after digestion.** The table below displays average peak areas (n = 3), and differences were evaluated to assess impact of clean-up stage (before vs. after digestion) on the analyte's signal intensity. The last column displays the % reduction of peak area for d-SPE clean-up after digestion as compared to no clean-up. *ND* = not detected.	S20
**Table S3. Results from pilot validation using ctDNA.** ctDNA was treated with Zsep+ (n=3) and the concentration was measured before and after the treatment. Recovery was calculated as % of DNA after treatment compared to before treatment, and reduction in DNA was calculated using the difference before and after treatment compared to before treatment.	S21
**Table S4. Results of the Wilcoxon paired test applied to assess the fold change in DNA adduct levels following clean-up treatment.** Columns show the median fold change, ranks (positive, negative, and signed), p-values using Pratt's method (two tailed) and associated statistical summaries. Significant changes are indicated with ** ($p < 0.01$) and * ($p < 0.05$), while non-significant results are marked as ns. Discrepancies and 95% confidence intervals are included to highlight the direction and magnitude of the fold change. Not affected adducts (A17 and A29) and statistically significant decreases (dI and dU) are indicated in bold, whereas most adducts exhibit positive changes.	S22

Notes

Note 1. LC-HRMS approach

A reverse phase UPLC column, Acquity HSS T3 column, was used for chromatographic separation of 2'-deoxyribonucleoside adducts. The 16 min LC gradient employed to elute the 2′-deoxyribonucleoside adducts provided sharp and symmetric chromatographic peaks. The optimized LC gradient program provided adequate chromatographic retention of all the detected analytes and sufficient equilibration time between consecutive runs. Based on the narrow peak widths (~ 12–15 sec) obtained by the UPLC-MS approach, it is possible to further reduce the LC run time to achieve high throughput of the analytical method whilst retaining adequate resolution of the diverse range of DNA adducts. The resolution employed in this method (60000 for full scan and 30000 for data-independent HRMS/MS scan) were judged to be appropriate settings; ~ 6–8 full scan data points for each MS1 chromatographic peak were obtained. The MS1 and MS2 ions detected at high mass accuracy (± 5 ppm mass error) significantly enhanced the confidence in DNA adduct detection and identification.

DIA mode of data acquisition involved fragmentation of ions spanning across a selected mass range (200–350 *m/z*) in different isolation windows. Thus, DIA is useful to provide a comprehensive, unbiased detection of the adduct analytes, in contrast to data-dependent acquisition (DDA), where ion intensity is used as a criterion for fragmentation and is prone to missing the detection of lower-level analytes. Although DDA would result in comparatively clean MS2 spectra, there is a risk of losing the information of the less abundant compounds. Since most of the DNA adducts are typically present in traces in complex biological matrices, we selected DIA following earlier approach for DNA adduct analysis.[10,15,39]

Note 2. DNA adductomics database

At the outset, the entries in the database were primarily limited to the adducts previously identified in *M. affinis*.[10,39] Thereafter, putative adducts from *nLossFinder* screening and GitLab data repository[17] were included. For the previously reported adducts in amphipods,[10,39] the MS raw data were first evaluated manually in Qual Browser of XCalibur 3.1 (Thermo Fisher Scientific) to detect 2'-deoxyribonucleoside adducts as protonated ions, *m/z* $[M+H]^+$ (MS1), and their corresponding nucleobase adduct ion (MS2) within the same chromatographic retention, using a mass accuracy of 5 ppm. Second, *nLossFinder* was run to screen for earlier unknown putative 2′-deoxyribonucleoside adducts from the MS raw data, which is based on matching peaks between the MS1 and MS2 spectra, using the characteristic neutral loss of deoxyribose (116.0473 Da). The tolerance for the neutral loss was set at 5 ppm, the criteria of minimum track points and track missing points were set as 3 and 1, respectively, and all other parameters were adopted as described earlier.[15] Third, an existing DNA adduct database[17] [https://gitlab.com/nexs-metabolomics/projects/dna_adductomics_database] was

imported to TraceFinder software (V4.1, Thermo Fisher Scientific), and the MS raw data were screened for putative 2′-deoxyribonucleoside adducts with 5 ppm accuracy of the protonated molecular ion and for the corresponding nucleobase adduct fragment ion. The outputs from these three steps were combined to create the in-house database (Table S1) of 2'-deoxyribonucleoside adducts relevant to amphipod DNA adductome. Each of the assigned putative adducts was included as a separate entry to the in-house database only after it was manually confirmed to be present in a pooled Quality Control sample (amphipod DNA digest from nine biological replicates). The candidates were filtered as possible nucleoside adducts when both the parent ion, $[M+H]^+$, and corresponding specific fragment ion (from dR loss, $[(M+H)\text{-}dR]^+$) were detected within the same chromatographic peak, for which the following equation sustained within a 5 ppm mass tolerance for each ion; $[M+H]^+ = [(M+H)\text{-}dR]^+ + 116.0473$.

Each adduct in the database (Table S1) has 3 entries (rows), one for the molecular ion (called TargetPeak, ms1 order) and two for the fragments (associated TargetPeak, ms2 order), all within the same chromatographic retention. One of the fragment entries corresponds to loss of deoxyribose $[(M+H)\text{-}dR]^+$ and the other fragment entry corresponds to the deoxyribose ion $[dR]^+$ (*m/z* 117.055).

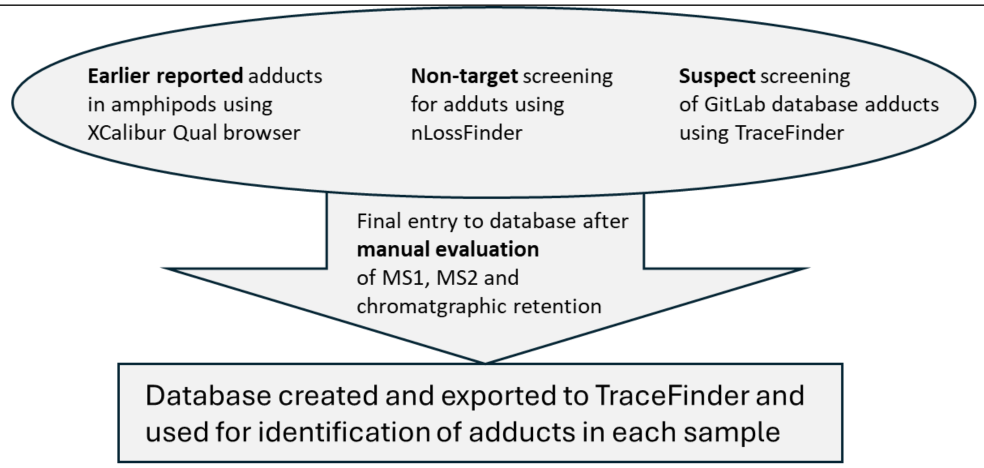

Box 1. Schematic representation of the steps involved in the generation and use of the database

Supplementary Figures

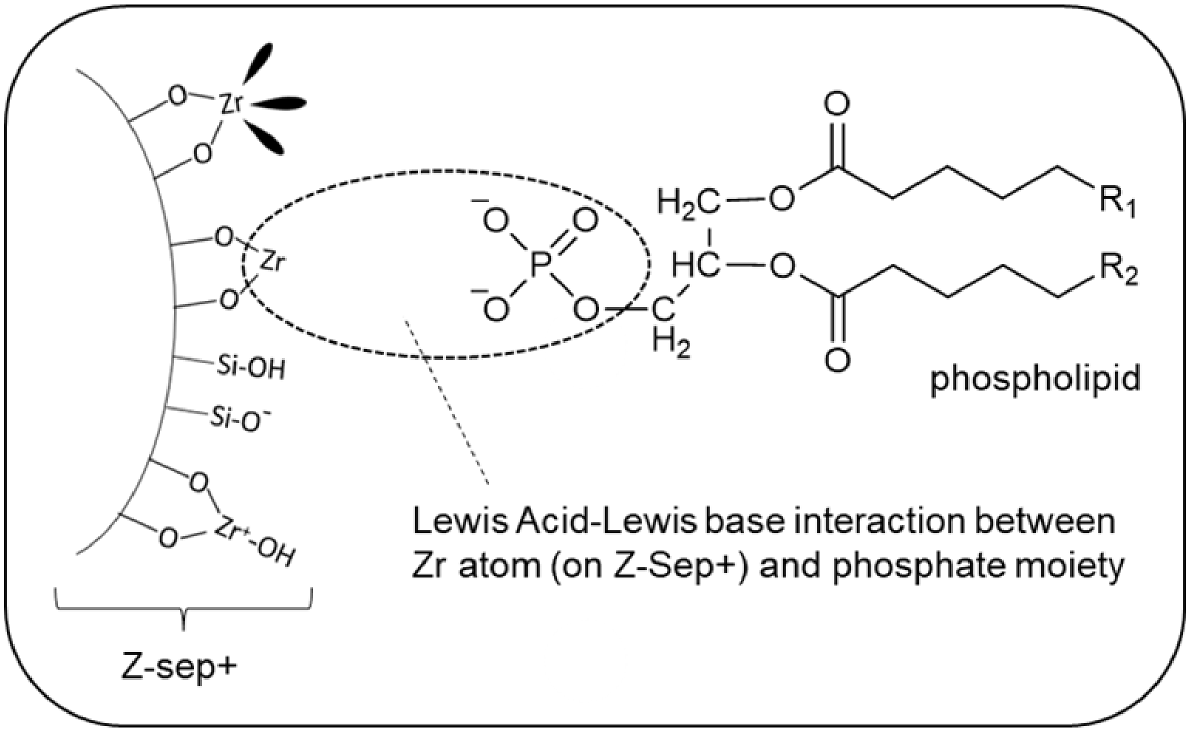

Fig. S1. Illustration of retention mechanism on Z-sep+ sorbent for matrix components exemplified with a phospholipid. Zirconia (Zr) coated on silica acts as a Lewis acid, while the phosphate group of phospholipids (or hydroxy moiety of fats, such as monoacylglycerol) act as a Lewis base during the interaction. Hydrophobic interaction involving C18 is not depicted here.

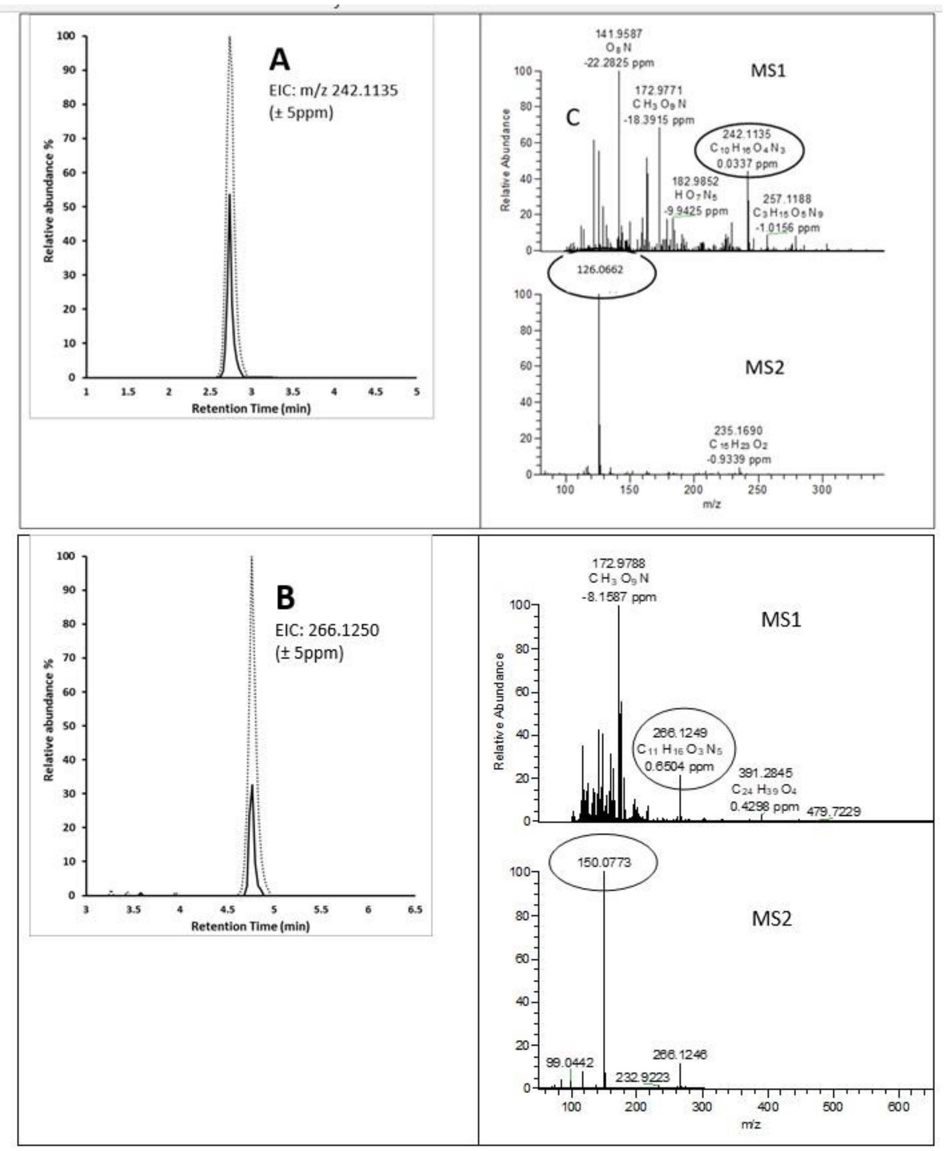

Fig. S2. Improved signal of 5-me-dC (A) and N^6-me-dA (B) employing d-SPE clean-up in amphipod DNA followed by their spectral identification with parent ion (MS1) and fragment ion (MS2), both within 5 ppm mass accuracy. In case of 5-me-dC, the EIC at observed *m/z* 242.1135 (± 5ppm) showing the signal in DNA extract processed without d-SPE clean-up (solid line) and the correspondingly enhanced signal intensity, approx. by 50% (dotted line) after use of d-SPE clean-up (dotted line) under similar processing conditions. Similarly, for N^6-me-dA the EIC at observed *m/z* 266.1250 (± 5ppm) was correspondingly enhanced by 70 % after d-SPE clean-up.

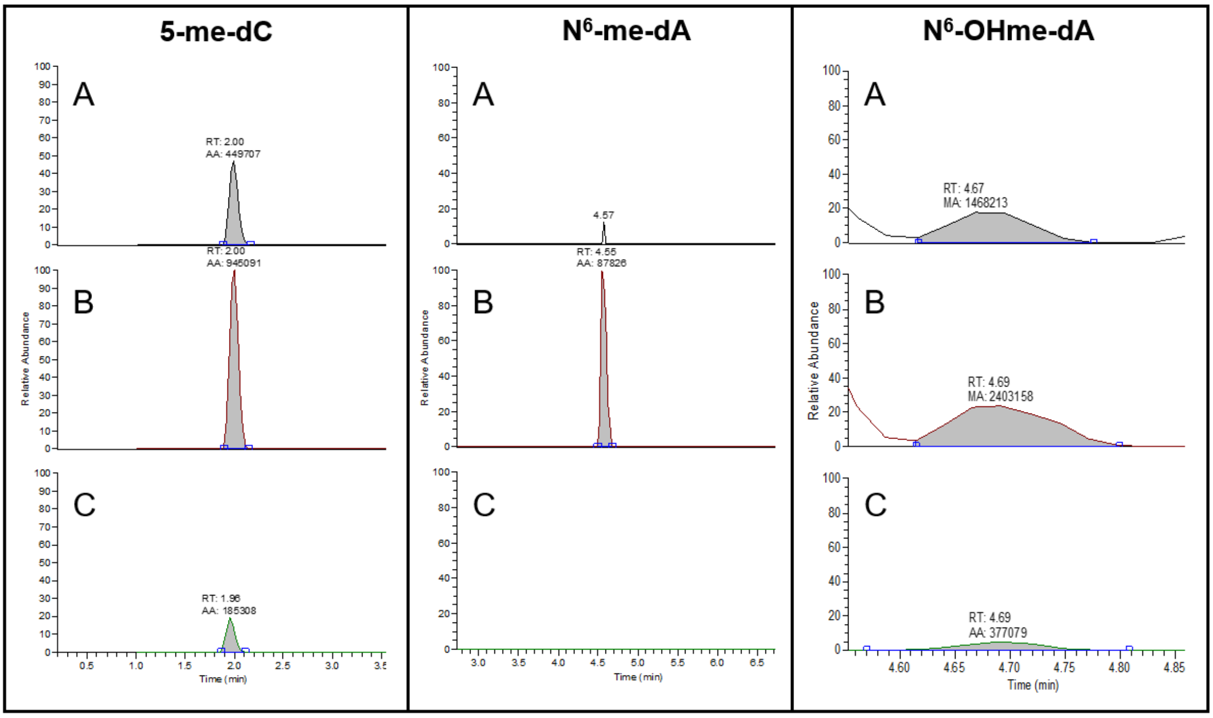

Fig. S3. Comparison of peak responses of 5-me-dC, N^6-me-dA and **N^6-OHme-dA in extracts with no clean-up (A); clean-up before digestion (B) and clean-up after digestion (C).** Retention time (Rt, min) and peak area (AA) are indicated on each chromatogram. Clean-up performed prior to digestion markedly enhanced peak intensity, consistent with removal of interfering matrix components before enzymatic hydrolysis. In contrast, clean-up after digestion resulted in notable signal loss for all analytes.

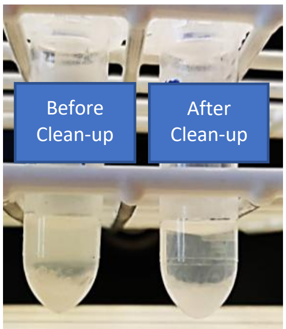

Fig. S4. Visual comparison of the amphipod DNA extract before (left) and after (right) d-SPE clean-up. The extract before clean-up, using 35 mg Z-sep+, was visibly more turbid compared to that after clean-up because of the potential removal of phospholipids, fats and pigment moieties from the extract.

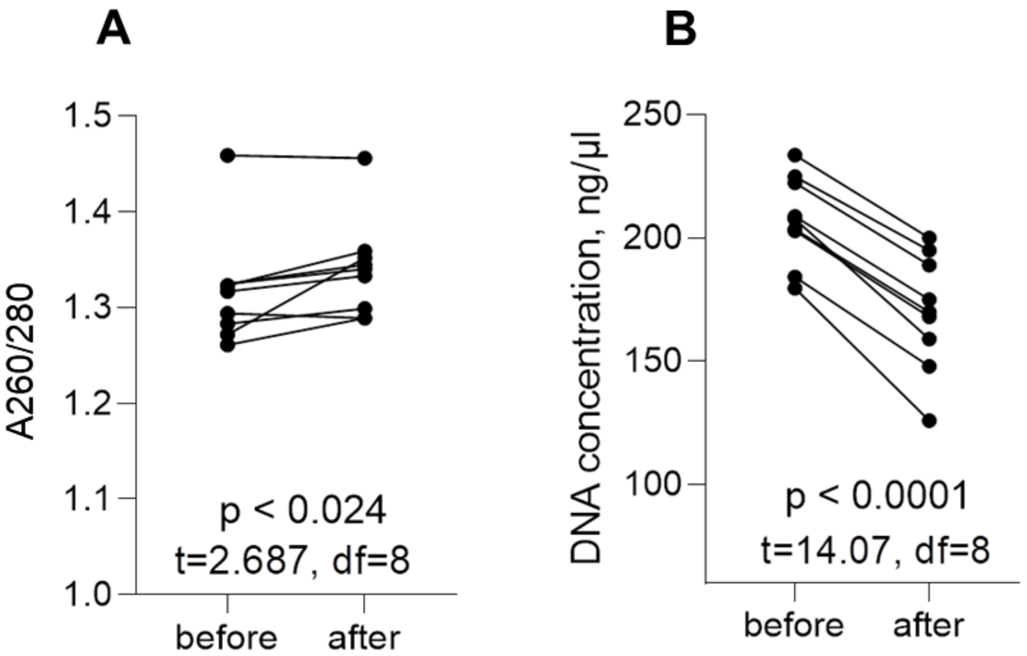

Fig. S5. Paired t-test results showing changes in (A) A260/280 purity ratio and (B) DNA concentration (ng/µL) before and after d-SPE clean-up. Significant increase in A260/280 ratio ($p < 0.024$, $t = 2.687$, $df = 8$) indicates improved sample purity, while significant decrease in DNA concentration ($p < 0.0001$, $t = 14.07$, $df = 8$) reflects removal of contaminants contributing to initial overestimation of DNA content.

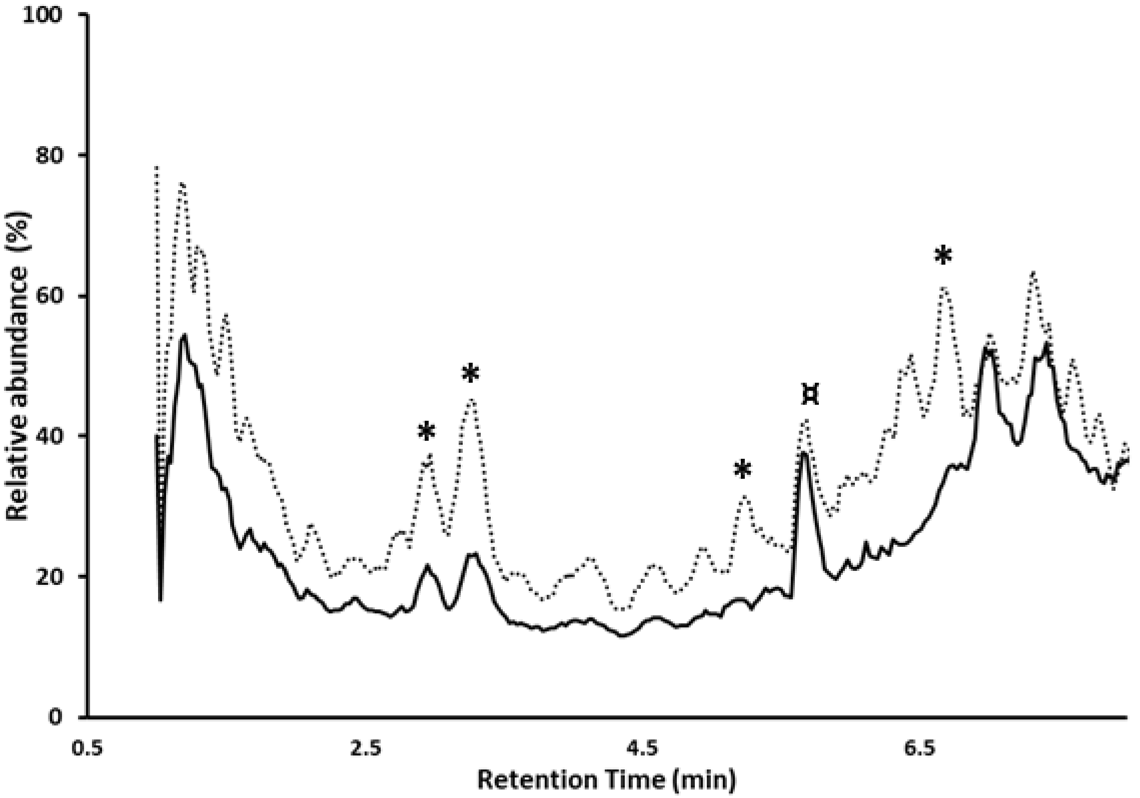

Fig. S6. A section of overlaid chromatograms of the amphipod DNA digest following d-SPE extraction with Z-sep+ (continuous line) and without d-SPE (dotted line) demonstrating the effect of the clean-up on the removal of matrix co-extractives. The relatively high intensity of numerous matrix background signals in the extract without clean-up could be effectively reduced after clean-up, especially at the retention time where most of the 2'-deoxyribonucleoside adducts elute (within the range of 2 to 7 min). Peaks representing peptides and a phospholipid are marked as * and ¤, respectively.

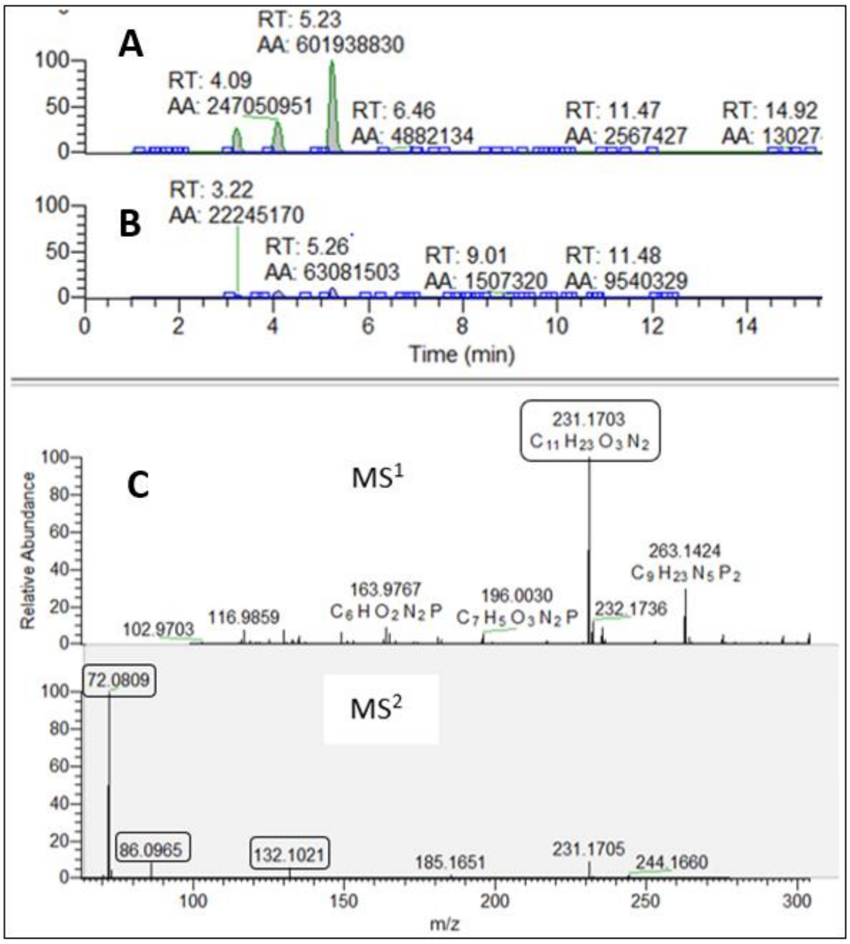

Fig. S7. d-SPE clean-up effect: Reduction of dipeptide signal from the matrix after optimized d-SPE clean-up. Extracted ion chromatogram for *m/z* 231.1703 (± 5 ppm) showing the signal intensity of dipeptide 'VL' at Rt 5.23 min without d-SPE clean-up (**A**) and the correspondingly reduced signal intensity of 'VL' after d-SPE clean-up (**B**); **C**: Identification of 'VL' with respect to parent ion (MS1, and chemical formula) and fragment ions (MS2).

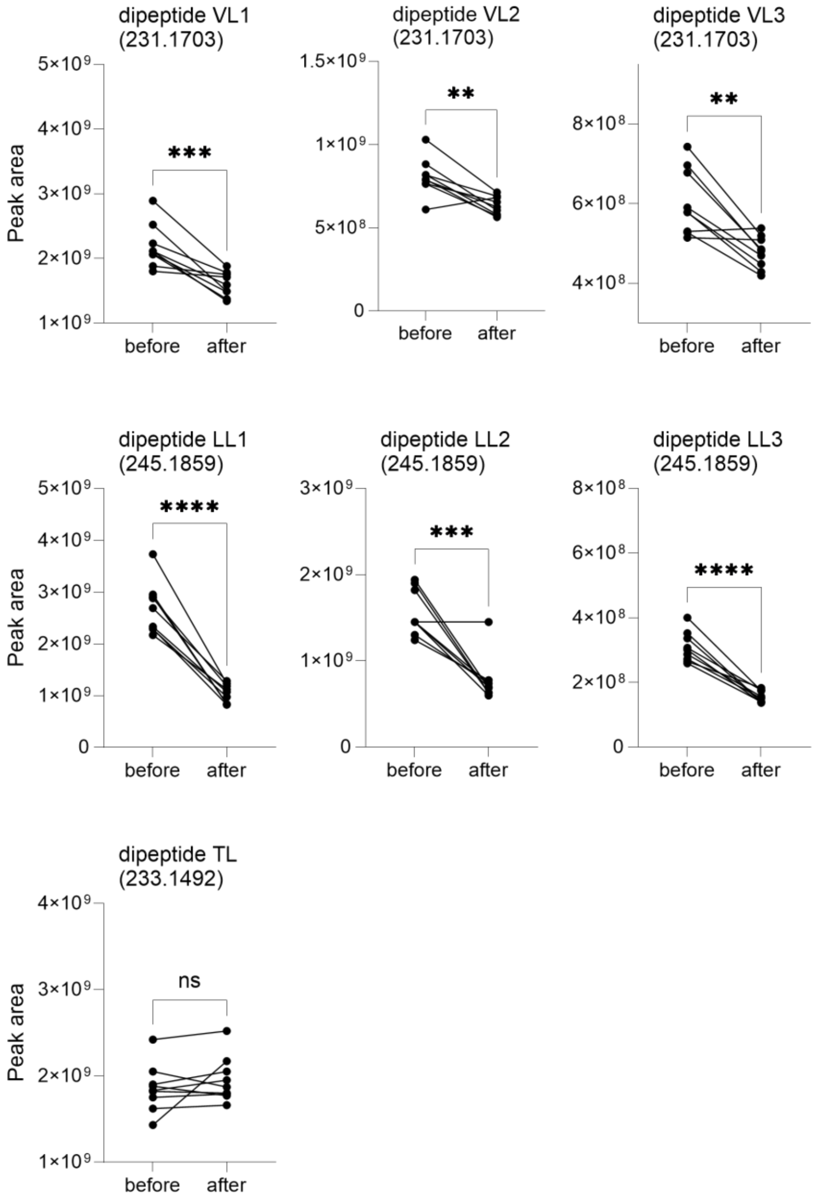

Fig. S8. Paired t-test results showing changes in peak areas for the dipeptides 'VL' 'LL' and 'TL' (*m/z* in parenthesis) before and after d-SPE clean-up as measured by LC-MS analysis. 'VL' and 'LL' exist as isomers at different Rt. Significant decrease in peak areas of these matrix compounds (p value summary; * =level of significance), except for TL, indicates sample purification as a result of d-SPE clean-up effect.

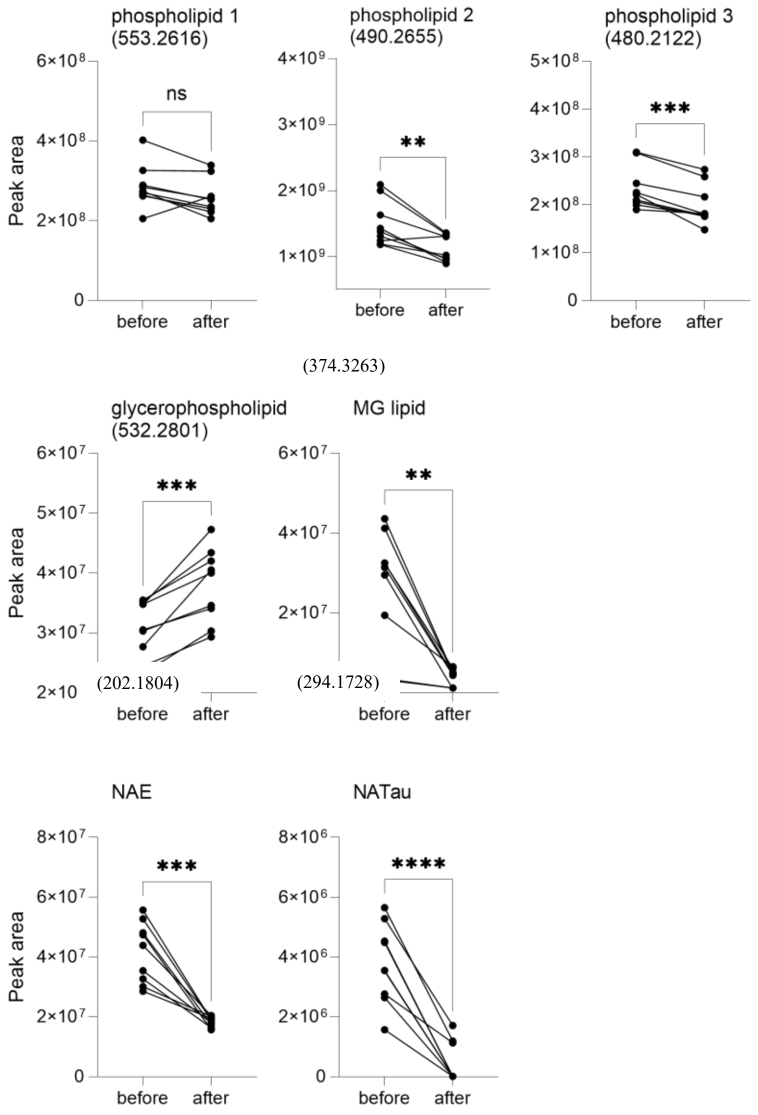

Fig. S9. Paired t-test results showing changes in peak areas for the phospholipid 1, phospholipid 2, phospholipid 3, glycerophospholipid, MG, NAE and NATau (*m/z* in parenthesis) before and after d-SPE clean-up as measured by LC-MS analysis. The lipids were identified based on LIPID MAPS and MS-DIAL data processing. Significant decrease in peak areas of these matrix compounds (p value summary; * =level of significance), except glycerophospholipid, indicates sample purification as a result of d-SPE clean-up effect.

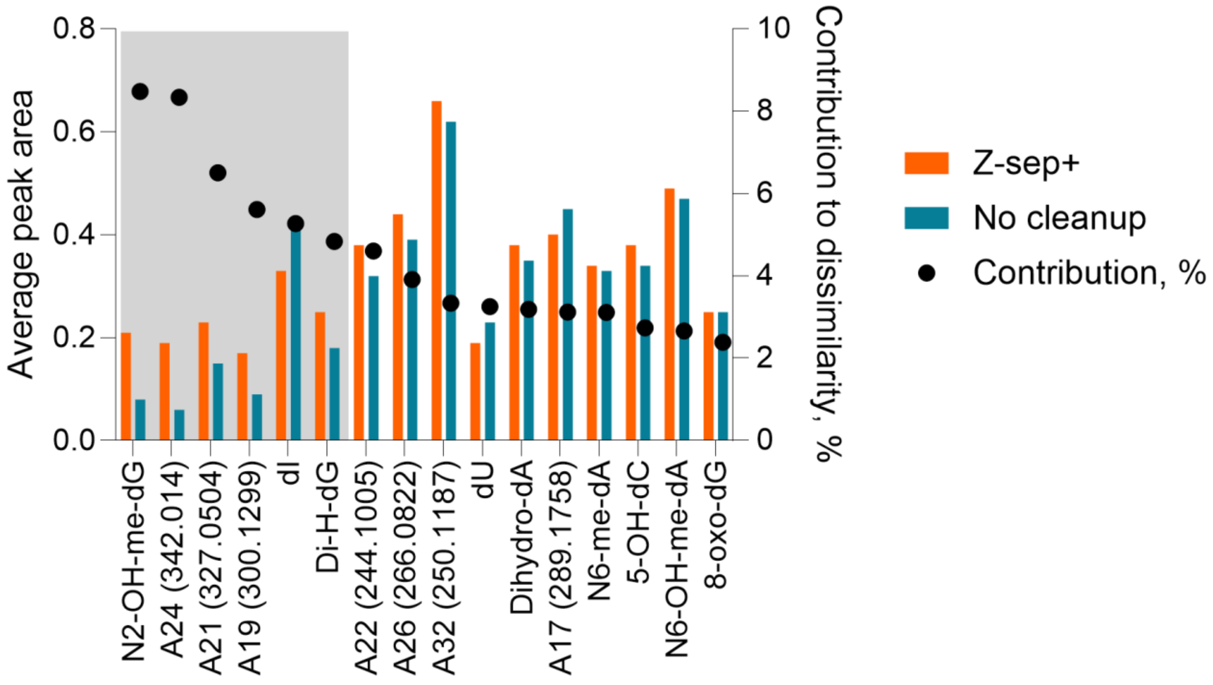

Fig. S10. Contributions of individual DNA adducts to the dissimilarity due to the treatment effect based on SIMPER analysis in the normalized dataset. Bar plot showing the average values of the most influential DNA adducts and their contribution (%) to the dissimilarity between the untreated samples (No clean-up, teal) and the same samples after the treatment (Z-sep+, orange); the adducts shown on this plot contributed 70% of the dissimilarity between the groups. Adducts in the grey-shaded area represent the most influential contributors to the treatment effect, with reduced levels (except dI) in the untreated group compared to the Z-sep+ group.

Supplementary Tables

Table S1. In-house DNA adduct database for screening of 2′-deoxyribonucleoside adducts in amphipods. Compound name for identified DNA adducts given as their standard abbreviation and for unidentified adducts as *m/z* of the parent ion. For each compound, the target peak (ms1 $[M+H]^+$), fragment peaks (ms2 $[(M+H)\text{-}dR]^+$ and $[dR]^+$) as well as retention time (min) are provided. A1-A19 (in the database table S1) are referring to adduct masses with the same A# as reported earlier in amphipods (Gorokhova et al, 2020).[39] They may not necessarily be the same number as that in Table 1.

Compound Name	Workflow	Associated Target Peak	MS Order	m/z	Retention Time (min)
**dC**	TargetPeak		ms1	228.0978	2.02
**dC**	Fragment	1	ms2	112.0505	2.02
**dC**	Fragment	1	ms2	117.0552	2.02
**dU**	TargetPeak		ms1	229.0818	3.8
**dU**	Fragment	1	ms2	113.0345	3.8
**dU**	Fragment	1	ms2	117.0552	3.8
**N4-OHme-5-medC**	TargetPeak		ms1	272.1241	1.9
**N4-OHme-5-medC**	Fragment	1	ms2	156.0768	1.9
**N4-OHme-5-medC**	Fragment	1	ms2	117.0552	1.9
**5,6-Di-H-dU**	TargetPeak		ms1	231.0976	2.81
**5,6-Di-H-dU**	Fragment	1	ms2	115.0503	2.81
**5,6-Di-H-dU**	Fragment	1	ms2	117.0552	2.81
**231.1337**	TargetPeak		ms1	231.1337	4.86
**231.1337**	Fragment	1	ms2	115.0864	4.86
**231.1337**	Fragment	1	ms2	117.0552	4.86
**239.059**	TargetPeak		ms1	239.059	9.7
**239.059**	Fragment	1	ms2	123.0117	9.7
**239.059**	Fragment	1	ms2	117.0552	9.7
**240.1593**	TargetPeak		ms1	240.1593	11.77
**240.1593**	Fragment	1	ms2	124.112	11.77
**240.1593**	Fragment	1	ms2	117.0552	11.77
**241.1074**	TargetPeak		ms1	241.1074	4.69
**241.1074**	Fragment	1	ms2	125.0601	4.69
**241.1074**	Fragment	1	ms2	117.0552	4.69
**5-me-dC**	TargetPeak		ms1	242.1136	2.6
**5-me-dC**	Fragment	1	ms2	126.0663	2.6
**5-me-dC**	Fragment	1	ms2	117.0552	2.6
**T**	TargetPeak		ms1	243.0974	5.42
**T**	Fragment	1	ms2	127.0501	5.42
**T**	Fragment	1	ms2	117.0552	5.42
**5-OH-dC**	TargetPeak		ms1	244.0936	5.4
**5-OH-dC**	Fragment	1	ms2	128.0463	5.4
**5-OH-dC**	Fragment	1	ms2	117.0552	5.4
**245.1591**	TargetPeak		ms1	245.1591	5.78
**245.1591**	Fragment	1	ms2	129.1118	5.78

Compound Name	Workflow	Associated Target Peak	MS Order	m/z	Retention Time (min)
**245.1591**	Fragment	1	ms2	117.0552	5.78
**248.1494**	TargetPeak		ms1	248.1494	5.59
**248.1494**	Fragment	1	ms2	132.1021	5.59
**248.1494**	Fragment	1	ms2	117.0552	5.59
**249.1096**	TargetPeak		ms1	249.1096	12.41
**249.1096**	Fragment	1	ms2	133.0623	12.41
**249.1096**	Fragment	1	ms2	117.0552	12.41
**250.1187**	TargetPeak		ms1	250.1187	11.7
**250.1187**	Fragment	1	ms2	134.0714	11.7
**250.1187**	Fragment	1	ms2	119.0606	11.7
**251.1187**	Fragment	2	ms3	117.0552	11.7
**251.0612**	TargetPeak		ms1	251.0612	1.58
**251.0612**	Fragment	1	ms2	135.0139	1.58
**251.0612**	Fragment	1	ms2	117.0552	1.58
**dA**	TargetPeak		ms1	252.1089	3.94
**dA**	Fragment	1	ms2	136.0616	3.94
**dA**	Fragment	1	ms2	117.0552	3.94
**dI (deoxyinosine)**	TargetPeak		ms1	253.0931	4.72
**dI (deoxyinosine)**	Fragment	1	ms2	137.0458	4.72
**dI (deoxyinosine)**	Fragment	1	ms2	117.0552	4.72
**Di-H-dA**	TargetPeak		ms1	254.1248	3.97
**Di-H-dA**	Fragment	1	ms2	138.0775	3.97
**Di-H-dA**	Fragment	1	ms2	117.0552	3.97
**5-OH-me-dC**	TargetPeak		ms1	258.1085	2.15
**5-OH-me-dC**	Fragment	1	ms2	142.0612	2.15
**5-OH-me-dC**	Fragment	1	ms2	117.0552	2.15
**261.1311**	TargetPeak		ms1	261.1311	5.95
**261.1311**	Fragment	1	ms2	145.0838	5.95
**261.1311**	Fragment	1	ms2	117.0552	5.95
**262.1185**	TargetPeak		ms1	262.1185	6.5
**262.1185**	Fragment	1	ms2	146.0712	6.5
**262.1185**	Fragment	1	ms2	117.0552	6.5
**262.1289**	TargetPeak		ms1	262.1289	5.26
**262.1289**	Fragment	1	ms2	146.0816	5.26
**262.1289**	Fragment	1	ms2	117.0552	5.26
**N6-me-dA**	TargetPeak		ms1	266.1257	4.6
**N6-me-dA**	Fragment	1	ms2	150.0784	4.6
**N6-me-dA**	Fragment	1	ms2	117.0552	4.6
**dG**	TargetPeak		ms1	268.1033	4.71
**dG**	Fragment	1	ms2	152.056	4.71
**dG**	Fragment	1	ms2	117.0552	4.71
**OH-ε-dC**	TargetPeak		ms1	270.109	4.71

Compound Name	Workflow	Associated Target Peak	MS Order	m/z	Retention Time (min)
**OH-ε-dC**	Fragment	1	ms2	154.0617	4.71
**OH-ε-dC**	Fragment	1	ms2	117.0552	4.71
**Di-H-dG**	TargetPeak		ms1	270.1197	4.8
**Di-H-dG**	Fragment	1	ms2	154.0724	4.8
**Di-H-dG**	Fragment	1	ms2	117.0552	4.8
**270.163**	TargetPeak		ms1	270.163	6.44
**270.163**	Fragment	1	ms2	154.1157	6.44
**270.163**	Fragment	1	ms2	117.0552	6.44
**Me-Glycol-dC**	TargetPeak		ms1	276.119	1.89
**Me-Glycol-dC**	Fragment	1	ms2	160.0717	1.89
**Me-Glycol-dC**	Fragment	1	ms2	117.0552	1.89
**279.0387**	TargetPeak		ms1	279.0387	2
**279.0387**	Fragment	1	ms2	162.9914	2
**279.0387**	Fragment	1	ms2	117.0552	2
**279.134**	TargetPeak		ms1	279.134	5.11
**279.134**	Fragment	1	ms2	163.0867	5.11
**279.134**	Fragment	1	ms2	117.0552	5.11
**N^6-OHme-dA**	TargetPeak		ms1	282.1202	5.55
**N^6-OHme-dA**	Fragment	1	ms2	166.0729	5.55
**N^6-OHme-dA**	Fragment		ms3	136.0617	5.55
**N^6-OHme-dA**	Fragment	1	ms2	117.0552	5.55
**283.1265**	TargetPeak		ms1	283.1265	3.35
**283.1265**	Fragment	1	ms2	167.0792	3.35
**283.1265**	Fragment	1	ms2	117.0552	3.35
**8-oxo-dG**	TargetPeak		ms1	284.0989	5.45
**8-oxo-dG**	Fragment	1	ms2	168.0516	5.45
**8-oxo-dG**	Fragment	1	ms2	117.0552	5.45
**288.1916**	TargetPeak		ms1	288.1916	4.94
**288.1916**	Fragment	1	ms2	172.1443	4.94
**288.1916**	Fragment	1	ms2	117.0552	4.94
**A7**	TargetPeak		ms1	289.1758	11.6
**A7**	Fragment	1	ms2	173.1285	11.6
**A7**	Fragment	1	ms2	117.0552	11.6
**290.0858**	TargetPeak		ms1	290.0858	4.73
**290.0858**	Fragment	1	ms2	174.0385	4.73
**290.0858**	Fragment	1	ms2	117.0552	4.73
**291.0891**	TargetPeak		ms1	291.0891	4.7
**291.0891**	Fragment	1	ms2	175.0418	4.7
**291.0891**	Fragment	1	ms2	117.0552	4.7
**292.129**	TargetPeak		ms1	292.129	7.15
**292.129**	Fragment	1	ms2	176.0817	7.15
**292.129**	Fragment	1	ms2	117.0552	7.15

Compound Name	Workflow	Associated Target Peak	MS Order	m/z	Retention Time (min)
**M1-dT**	TargetPeak		ms1	297.1081	4.94
**M1-dT**	Fragment	1	ms2	181.0608	4.94
**M1-dT**	Fragment	1	ms2	117.0552	4.94
**N2-OH-Me-dG**	TargetPeak		ms1	298.1146	3.93
**N2-OH-Me-dG**	Fragment	1	ms2	182.0673	3.93
**N2-OH-Me-dG**	Fragment	1	ms2	117.0552	3.93
**A12**	TargetPeak		ms1	300.1305	3.82
**A12**	Fragment	1	ms2	184.0832	3.82
**A12**	Fragment	1	ms2	117.0552	3.82
**A12_1**	TargetPeak		ms1	300.1305	4.73
**A12_1**	Fragment	1	ms2	184.0832	4.73
**A12_1**	Fragment	1	ms2	117.0552	4.73
**N4-(4-OHbut)-dC**	TargetPeak		ms1	300.1552	6.24
**N4-(4-OHbut)-dC**	Fragment	1	ms2	184.1079	6.24
**N4-(4-OHbut)-dC**	Fragment	1	ms2	117.0552	6.24
**302.1341**	TargetPeak		ms1	302.1341	3.44
**302.1341**	Fragment	1	ms2	186.0868	3.44
**302.1341**	Fragment	1	ms2	117.0552	3.44
**A17**	TargetPeak		ms1	306.0595	3.95
**A17**	Fragment	1	ms2	190.0122	3.95
**A17**	Fragment	1	ms2	117.0552	3.95
**308.1273**	TargetPeak		ms1	308.1273	3.76
**308.1273**	Fragment	1	ms2	192.08	3.76
**308.1273**	Fragment	1	ms2	117.0552	3.76
**309.1313**	TargetPeak		ms1	309.1313	3.72
**309.1313**	Fragment	1	ms2	193.084	3.72
**309.1313**	Fragment	1	ms2	117.0552	3.72
**311.0728**	TargetPeak		ms1	311.0728	5.38
**311.0728**	Fragment	1	ms2	195.0255	5.38
**311.0728**	Fragment	1	ms2	117.0552	5.38
**Carboxy-OH-Et-dC**	TargetPeak		ms1	316.1139	2.34
**Carboxy-OH-Et-dC**	Fragment	1	ms2	200.0666	2.34
**Carboxy-OH-Et-dC**	Fragment	1	ms2	117.0552	2.34
**318.1659**	TargetPeak		ms1	318.1659	5.61
**318.1659**	Fragment	1	ms2	202.1186	5.61
**318.1659**	Fragment	1	ms2	117.0552	5.61
**318.1659_1**	TargetPeak		ms1	318.1659	5.87
**318.1659_1**	Fragment	1	ms2	202.1186	5.87
**318.1659_1**	Fragment	1	ms2	117.0552	5.87
**319.1401**	TargetPeak		ms1	319.1401	7.21
**319.1401**	Fragment	1	ms2	203.0928	7.21
**319.1401**	Fragment	1	ms2	117.0552	7.21

Compound Name	Workflow	Associated Target Peak	MS Order	m/z	Retention Time (min)
**A19**	TargetPeak		ms1	327.0496	4.7
**A19**	Fragment	1	ms2	211.0023	4.7
**A19**	Fragment	1	ms2	117.0552	4.7
**328.1505**	TargetPeak		ms1	328.1505	5.27
**328.1505**	Fragment	1	ms2	212.1032	5.27
**328.1505**	Fragment	1	ms2	117.0552	5.27
**328.19**	TargetPeak		ms1	328.19	8.15
**328.19**	Fragment	1	ms2	212.1427	8.15
**328.19**	Fragment	1	ms2	117.0552	8.15
**330.2077**	TargetPeak		ms1	330.2077	5.8
**330.2077**	Fragment	1	ms2	214.1604	5.8
**330.2077**	Fragment	1	ms2	117.0552	5.8
**330.2384**	TargetPeak		ms1	330.2384	7.8
**330.2384**	Fragment	1	ms2	214.1911	7.8
**330.2384**	Fragment	1	ms2	117.0552	7.8
**332.1814**	TargetPeak		ms1	332.1814	5.34
**332.1814**	Fragment	1	ms2	216.1341	5.34
**332.1814**	Fragment	1	ms2	117.0552	5.34
**pentenal-dA**	TargetPeak		ms1	336.1666	4.68
**pentenal-dA**	Fragment	1	ms2	220.1193	4.68
**pentenal-dA**	Fragment	1	ms2	117.0552	4.68
**339.1988**	TargetPeak		ms1	339.1988	5.95
**339.1988**	Fragment	1	ms2	223.1515	5.95
**339.1988**	Fragment	1	ms2	117.0552	5.95
**342.0143**	TargetPeak		ms1	342.0143	5.42
**342.0143**	Fragment	1	ms2	225.967	5.42
**342.0143**	Fragment	1	ms2	117.0552	5.42
**343.7132**	TargetPeak		ms1	343.7132	6.24
**343.7132**	Fragment	1	ms2	227.6659	6.24
**343.7132**	Fragment	1	ms2	117.0552	6.24
**348.1558**	TargetPeak		ms1	348.1558	4.48
**348.1558**	Fragment	1	ms2	232.1085	4.48
**348.1558**	Fragment	1	ms2	117.0552	4.48
**319.1694**	TargetPeak		ms1	319.1694	6.83
**319.1694**	Fragment	1	ms2	203.1221	6.83
**319.1694**	Fragment	1	ms2	117.0552	6.83
**Heptenal-dC**	TargetPeak		ms1	340.1872	5.5
**Heptenal-dC**	Fragment	1	ms2	224.1349	5.5
**Heptenal-dC**	Fragment	1	ms2	298.1397	5.5
**Heptenal-dC**	Fragment	1	ms2	117.0552	5.5
**5,6-Di-H-dT**	TargetPeak		ms1	245.1137	4.35
**5,6-Di-H-dT**	Fragment	1	ms2	129.0664	4.35

Compound Name	Workflow	Associated Target Peak	MS Order	m/z	Retention Time (min)
**5,6-Di-H-dT**	Fragment	1	ms2	117.0552	4.35
**239.1139**	TargetPeak		ms1	239.1139	3.2
**239.1139**	Fragment	1	ms2	123.0667	3.2
**239.1139**	Fragment	1	ms2	117.0552	3.2
**244.1005**	TargetPeak		ms1	244.1005	4.7
**244.1005**	Fragment	1	ms2	128.0535	4.7
**244.1005**	Fragment	1	ms2	117.0552	4.7
**254.1151**	TargetPeak		ms1	254.1151	3.2
**254.1151**	Fragment	1	ms2	138.068	3.2
**254.1151**	Fragment	1	ms2	117.0552	3.2
**231.1339**	TargetPeak		ms1	231.1339	1.8
**231.1339**	Fragment	1	ms2	115.0867	1.8
**231.1339**	Fragment	1	ms2	117.0552	1.8
**266.0822**	TargetPeak		ms1	266.0822	4.75
**266.0822**	Fragment	1	ms2	150.0357	4.75
**266.0822**	Fragment	1	ms2	117.0552	4.75
**253.1117**	TargetPeak		ms1	253.1117	3.2
**253.1117**	Fragment	1	ms2	137.0647	3.2
**253.1117**	Fragment	1	ms2	117.0552	3.2

Table S2. Comparison of peak responses (absolute signal intensities) of representative nucleosides and DNA adducts in samples with no clean-up, d-SPE clean-up before digestion, and d-SPE clean-up after digestion. The table below displays average peak responses (n = 3), and differences were evaluated to assess impact of clean-up stage (before vs. after digestion) on the analyte's signal intensity. The last column displays the % reduction of peak area for d-SPE clean-up after digestion as compared to no clean-up. *ND* = not detected.

Short name	No clean-up	d-SPE clean-up before digestion	d-SPE clean-up after digestion	% reduction
**dC**	6.31E+07	1.40E+08	3.23E+07	48.81
**dG**	1.83E+08	3.28E+08	3.97E+07	78.31
**5-me-dC**	1.35E+06	3.30E+06	4.43E+05	67.19
**N6-me-dA**	7.25E+04	1.96E+05	*ND*	*ND*
**dI**	5.74E+06	3.91E+06	1.65E+06	71.25
**dU**	7.15E+05	5.86E+05	2.39E+05	66.57
**N6-OH-me-dA**	1.33E+06	3.00E+06	3.70E+05	72.18

Table S3. Results from pilot validation using ctDNA. ctDNA was treated with Zsep+ (n=3) and the concentration was measured before and after the treatment. Recovery was calculated as % of DNA after treatment compared to before treatment, and reduction in DNA was calculated using the difference before and after treatment compared to before treatment.

	Concentration (ng/uL)			Mean conentration (ng/uL)	%-Recovery	%-Reduction	P value (t-test)
ctDNA untreated	314.7	327.6	324.5	322.2	-	-	-
after Zsep+ (1)	309.2	317.5	314.6	313.8	97	2.6	0.152
after Zsep+ (2)	305.2	307.3	311.9	308.1	96	4.4	0.049
after Zsep+ (3)	305.0	305.9	302.0	304.3	94	5.6	0.035

Table S4. Results of the Wilcoxon paired test applied to assess the fold change in DNA adduct levels following clean-up treatment. Columns show the median fold change, ranks (positive, negative, and signed), p-values using Pratt's method (two tailed) and associated statistical summaries. Significant changes are indicated with ** ($p < 0.01$) and * ($p < 0.05$), while non-significant results are marked as ns. Discrepancies and 95% confidence intervals are included to highlight the direction and magnitude of the fold change. Not affected adducts (A17 and A29) and statistically significant decreases (dI and dU) are indicated in bold, whereas most adducts exhibit positive changes.

DNA Adduct	Actual median	Sum of signed ranks	Sum of positive ranks	Sum of negative ranks	P value	P value summary	Discrepancy	95% confidence in terval
5-me-dC	1.7	45	45	0	0	**	0.67	0.6400 to 0.8400
N^6-me-dA	1.7	45	45	0	0	**	0.67	0.3900 to 1.020
8-oxo-dG	1.5	43	44	-1	0.01	**	0.52	0.05000 to 0.7600
5-OH-dC	2.2	45	45	0	0	**	1.23	0.6700 to 2.980
**dI**	**0.6**	**-45**	**0**	**-45**	**0**	**	**-0.43**	**-0.5210 to -0.1110**
**dU**	**0.7**	**-35**	**5**	**-40**	**0.04**	*	**-0.26**	**-0.7170 to 0.05000**
N^4-OH-me-5-medC	1.4	45	45	0	0	**	0.37	0.1300 to 0.9800
Me-Glycol-dC	1.6	45	45	0	0	**	0.56	0.1700 to 1.060
OH-ε-dC	1.4	45	45	0	0	**	0.44	0.1800 to 0.7800
Heptenal-dC	1.2	45	45	0	0	**	0.23	0.1500 to 0.5300
Dihydro-dA	2.2	45	45	0	0	**	1.15	0.1000 to 1.880
N^4-(4-OH-But)-dC	1.3	45	45	0	0	**	0.29	0.1500 to 0.4600
Di-H-dG	3.6	45	45	0	0	**	2.56	0.7400 to 210.0
N^2-OH-me-dG	626	45	45	0	0	**	625	1.710 to 2289
5,6-Di-H-dT	1.3	45	45	0	0	**	0.3	0.1700 to 0.4400
N^6-OH-me-dA	1.6	45	45	0	0	**	0.61	0.5300 to 1.170
**A17 (289.1758)**	**0.9**	**-21**	**12**	**-33**	**0.25**	**ns**	**-0.06**	**-0.1600 to 0.03000**
A18 (239.1139)	1.4	45	45	0	0	**	0.38	0.1300 to 0.7800
A19 (300.1299)	113	45	45	0	0	**	112	1.520 to 236.0
A20 (306.0603)	1.6	43	44	-1	0.01	**	0.57	0.2900 to 1.590

DNA Adduct	Actual median	Sum of signed ranks	Sum of positive ranks	Sum of negative ranks	P value	P value summary	Discrepancy	95% confidence in terval
A21 (327.0504)	103	39	42	-3	0.02	*	102	-0.09500 to 136.0
A22 (244.1005)	2.6	45	45	0	0	**	1.58	0.6700 to 10.30
A23 (254.1151)	1.7	45	45	0	0	**	0.71	0.3600 to 1.290
A24 (342.014)	143	45	45	0	0	**	142	103.0 to 220.0
A25 (231.1339)	1.5	43	44	-1	0.01	**	0.47	0.07000 to 0.5500
A26 (266.0822)	2.6	45	45	0	0	**	1.61	0.7300 to 2.900
A27 (261.1302)	1.4	39	42	-3	0.02	*	0.4	0.1100 to 0.4300
A28 (253.1117)	1.4	45	45	0	0	**	0.38	0.1300 to 0.6600
**A29 (240.1593)**	**1**	**-23**	**11**	**-34**	**0.2**	**ns**	**-0.03**	**-0.2430 to 0.06000**
A30 (270.1632)	1.3	45	45	0	0	**	0.29	0.1300 to 0.3700
A31 (288.1916)	1.4	45	45	0	0	**	0.43	0.1100 to 0.5400
A32 (250.1187)	1.7	45	45	0	0	**	0.74	0.4600 to 1.830
dA	1.5	45	45	0	0	**	0.51	0.3500 to 1.030
dG	1.5	45	45	0	0	**	0.45	0.3900 to 0.6900
dC	1.6	45	45	0	0	**	0.56	0.4800 to 0.9400
T	2	45	45	0	0	**	0.96	0.6200 to 1.810
